# Advancing Solvent Dehydration with Innovative HybSi^®^ AR Membranes: Economic and Environmental Benefits of Pervaporation

**DOI:** 10.3390/membranes15120367

**Published:** 2025-12-01

**Authors:** Mohammed Nazeer Khan, Elmar Boorsma, Pieter Vandezande, Ilse Lammerink, Rob de Lange, Anita Buekenhoudt, Miet Van Dael

**Affiliations:** 1 Unit Materials & Chemistry (MatCh), Flemish Institute for Technological Research (VITO), Boeretang 200, 2400 Mol, Belgiummiet.vandael@vito.be (M.V.D.); 2 Pervatech B.V., 7463 Rijssen, The Netherlandsrob.delange@aswyn.nl (R.d.L.); 3 Aswyn, Engelse Schans 12, 7137 SE Lievelde, The Netherlands; 4 Centre for Environmental Sciences (CMK), Hasselt University, Agoralaan, 3590 Diepenbeek, Belgium

**Keywords:** pervaporation, techno-economic assessment, solvent recovery, solvent dehydration, CO_2_ reduction, azeotropic distillation, extractive distillation

## Abstract

A techno-economic and environmental evaluation of dehydrating five industrially relevant solvents (isopropanol, acetonitrile, tetrahydrofuran, acetic acid, and n-methyl-2-pyrrolidone) using pervaporation-based processes was performed and compared to their respective traditional distillation processes. A standalone pervaporation and two hybrid processes (i.e., distillation-pervaporation and distillation-pervaporation-distillation) employing HybSi^®^ AR membranes were simulated in Aspen Plus, where the pervaporation module was modeled as a separator block that followed the experimental data. The experiments were performed at a vacuum pressure of 20 mbar and a temperature of 130 °C. The performance was compared based on several technical, economic, and environmental measures, of which key metrics are the levelized cost of separation (LCOS) and CO_2_ footprint reduction. From the economic perspective, the pervaporation-based processes are much more economical than the distillation processes for isopropanol (up to 42% reduction in LCOS) and acetonitrile (up to 39% reduction in LCOS), while their economic performance is similar to the benchmark process in the case of tetrahydrofuran (only up to 4% reduction in LCOS). For acetic acid (9% higher LCOS) and n-methyl-2-pyrrolidone (124% higher LCOS), the pervaporation-based processes do not perform better than the distillation processes under the current technical and economic considerations. However, a sensitivity analysis showed the potential to make the pervaporation-based processes more economical by improving the permeate flux and membrane module cost. On the other hand, the pervaporation-based processes are much more environmentally friendly for all the solvents studied compared to their respective benchmark processes. The reduction in CO_2_ footprint is in the order of 86%, 82%, 73%, 82%, and 65%, respectively, for the aforementioned solvents.

## 1. Introduction

Almost all industrial processes rely on organic solvents, with abundant use in a broad range of industries, from pharma and fine chemicals, over food, nutraceuticals, and cosmetics, to biofuels and paints [1]. In all these industries, solvents serve a variety of functions in the manufacture of essential chemicals and materials in our daily lives. However, the life cycles of industrial solvents, comprising production, transportation, use, and finally disposal, introduce sources of emissions that potentially impact human health and the environment in which they are released. Conventional solvent waste handling methods, especially incineration, are accompanied by significant increases in the overall energy, ecological, and safety footprint of solvent-intensive industries [2]. The continuously growing demand for solvents worldwide necessarily leads to higher levels of waste generation and higher waste disposal costs as well. Therefore, process intensification strategies are being considered to mitigate the growing costs as well as environmental, health, and safety concerns connected with waste solvent handling, prompted also by evolving regulatory frameworks promoting environmentally sound waste management methods.

Especially, solvent recovery is a major objective in the industries mentioned above to reduce production costs and improve the sustainability and circularity of current manufacturing practices. Thermal energy-based separation processes, such as distillation, are widely applied due to their exceptional performance in delivering high-purity streams [3]. However, these energy-intensive processes result in high operating costs and carbon emissions. The energy required for thermal separations is usually derived from steam produced by burning fossil fuels. In the case of solvents that form an azeotrope with another compound or have close boiling points, the energy consumption is even higher. Moreover, global energy costs are expected to increase significantly owing to the decline in the use of fossil fuels and the 2050 climate goals [4]. Using membranes either standalone or as hybrid systems has the potential to reduce energy consumption and CO_2_ emissions of thermal solvent recovery and dehydration processes significantly. Highly selective and energy-efficient pervaporation-based processes can reduce capital costs and energy consumption of traditional distillation processes by up to 40% while having substantial reductions in CO_2_ emissions [5].

In pervaporation, liquid mixtures are separated at the molecular level by the vaporization of target compounds through a selective membrane. The process has been coined “pervaporation” because of the unique co-occurring phenomena of “permeation” and “evaporation”, i.e., permselective transport and vaporization of molecules diffusing through the membrane [6]. The driving force for transport is a gradient in partial vapor pressure of the permeants between the liquid feed and vaporous permeate sides of the membrane. In practice, a vacuum is usually maintained at the permeate side. Since different compounds permeate at different rates, a substance at low concentration in the feed stream can be significantly enriched in the permeate. Pervaporation is generally employed when the target compounds are small, (semi-) volatile, and present at relatively low concentrations.

Despite some obvious similarities, some features make pervaporation significantly more efficient than distillation. Most notably, pervaporation is not governed by vapor-liquid equilibria but solely by the water/solvent separation factor of the membrane [7]. Whereas distillation is entirely determined by the thermodynamic equilibrium between the vapor and liquid phases, pervaporation predominantly relies on the affinity of the target molecules for the membrane [6]. This means that the permeate composition is not defined by the vapor-liquid equilibrium (VLE) but by the permeability of the compounds, which depends on their solubility and diffusion rate in the membrane. Hence, by using highly selective membranes, mixtures can be separated irrespective of the boiling points of the liquids and the occurrence of azeotropes. Since solute transport is dependent on the solubility of the penetrants in the membrane, the latter is chosen such that it interacts intensely with the target compounds. In the case of dehydration of organic solvents, hydrophilic membranes based on polymers, ceramics, or zeolites are being used [8,9]. Moreover, contrary to distillation involving repeated vaporization and condensation of the entire mixture, pervaporation only requires vaporization of the permeating fraction.

Pervaporation can be used as a standalone process, but owing to their modularity, membrane units can also be easily retrofitted to existing distillation plants. Such hybrid separations, where distillation and pervaporation are synergistically used, offer wide opportunities for debottlenecking and intensification of thermal separation processes [7]. This way, pervaporation may allow for significant reductions in energy requirements, often coupled with flexible increases in capacity and productivity, as well as improvements in raw material usage and reductions in waste volumes [7]. Hybrid distillation-pervaporation processes are being implemented in the industry mainly for dehydration and separation of solvents close to and beyond the azeotropic point, often as a cost-effective alternative for azeotropic or extractive distillation. Whereas distillation of mixtures with an azeotropic composition or with components with low relative volatility or close boiling points is energetically expensive, and auxiliary substances are usually required, pervaporation is performed in the absence of any additives, hence avoiding the need for extra recovery columns while eliminating the risk of product contamination.

The pervaporation membrane market is set to grow significantly, driven by the demand for solvent recovery and the industry’s need to reduce carbon footprints and reuse raw materials. Valued at 4.8 billion euros in 2023, the market is expected to grow at a CAGR of 6.9% until 2031 [10]. The HybSi^®^ AR membrane developed and commercialized by Pervatech is based on hybrid silica [5]. It is a robust, acid-resistant ceramic pervaporation membrane, which separates water and small polar compounds from various organic solvents and process mixtures [11]. It is used in industries such as chemical, pharmaceutical, food, and biotech for the dehydrating process and waste streams. HybSi^®^ AR offers superior chemical stability, higher operating temperatures, and a greater water/solvent separation factor, leading to significant operational flexibility and enhanced energy savings in dehydrating aggressive solvent streams [5,12]. Pervatech has already implemented a full-scale isopropanol (IPA) dehydration plant using these HybSi^®^ AR membranes [5]. More information on the advantages of these hybrid silica membranes can be found elsewhere [13,14].

In this study, we present a comprehensive techno-economic and environmental assessment of pervaporation-based solvent dehydration processes using HybSi^®^ AR membranes, applied across five industrially relevant solvent–water systems: isopropanol (IPA), acetonitrile (ACN), tetrahydrofuran (THF), acetic acid (ACA), and N-methyl-2-pyrrolidone (NMP). A variety of advanced distillation processes are commonly used for the dehydration of these solvents, including azeotropic, extractive, pressure-swing, and vacuum distillation. Many papers focus on the dehydration of IPA [15,16,17,18,19,20,21], ACN [22,23,24,25], THF [26,27,28,29,30,31,32], ACA [33,34,35,36,37,38], and NMP [39,40,41,42] by pervaporation, predominantly using novel laboratory-made membranes; however, a comprehensive techno-economic and environmental evaluation and benchmarking against distillation has never been reported. Distinct from previous works that often focus on individual solvents or simulated systems, this study integrates experimentally validated membrane performance data with Aspen Plus simulations to evaluate multiple hybrid process configurations, including D-PV, D-PV-D, and standalone PV, in comparison with traditional distillation-based benchmarks. Although the HybSi^®^ AR membrane itself is commercially available, its innovative use in high-temperature hybrid configurations for solvent dehydration, combined with experimentally validated performance data and process-level sustainability assessment, represents a significant contribution. In this context, the term ‘innovative’ in the title refers to the novel application and integration of the membrane, rather than the development of a new material. A novel contribution of this work is the introduction of a unified decision-making index, COPCO (COst savings per unit tonne of CO_2_ reduction), which enables simultaneous interpretation of both economic and environmental performance. By mapping each configuration within a cost-emission tradeoff space, we provide actionable guidance for solvent-specific process selection. This approach not only strengthens the case for pervaporation as a process intensification strategy but also offers a generalizable framework to support sustainable process design in solvent recovery. The insights obtained from this study are expected to further push the market uptake of pervaporation technology, particularly using HybSi^®^ AR membranes, and provide cost-efficient solutions to various pertinent dehydration challenges across solvent-intensive industries.

## 2. Materials and Methods

The following sections present the details of the pervaporation experiments and process modeling of the benchmark and the three pervaporation-based processes for all solvent-water systems under investigation. The selection of benchmark processes is guided by industry standards and supported by recommendations found in the literature [15,43,44,45,46]. The benchmark processes are discussed in detail in Section 2.2. Various types of distillation processes are incorporated as benchmarks in this study, as shown in Table 1. Of the three pervaporation-based processes, two are hybrid cases (distillation-pervaporation, D-PV, and distillation-pervaporation-distillation, D-PV-D), and the other is a standalone pervaporation (PV). Each solvent-water system has a benchmark and three pervaporation-based process configurations. A constant feed flow rate of 1000 kg/h at 20 °C and 1 atm was considered, and the feed has an equal composition of solvent and water in a 1:1 ratio by weight for all cases. The target purity of the dehydrated solvent is kept constant at 99.5 wt.% for all cases.

### 2.1. Pervaporation Experiments

**Figure 1:**
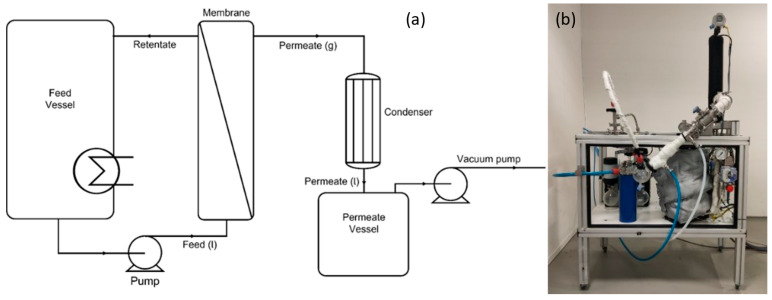
(**a**) Schematic overview of the lab-scale pervaporation equipment, (**b**) Photograph of the experimental laboratory apparatus.

A schematic overview of the equipment used for the pervaporation experiments is shown in Figure 1. Pervaporation experiments were performed with a set of four HybSi^®^ AR membranes with a total membrane area of 0.042 m^2^ for all five solvent-water systems. These were selected for their availability and suitability to enable consistent comparative evaluation across all tested solvent–water systems. For each experiment, a feed mixture was prepared containing one of the five solvents IPA (technical grade, Boom, Meppel, The Netherlands), ACN (HPLC-S gradient grade, Biosolve Chimie SARL, Valkenswaard, The Netherlands), THF (>99.9%, Sigma-Aldrich, Amsterdam, The Netherlands), ACA (99–100%, Boom, The Netherlands), and NMP (99%, Sigma-Aldrich, Amsterdam, The Netherlands) and reverse osmosis (RO) water. The feed vessel was filled with approximately 1.5–2 kg of the prepared mixture. The mixture was circulated over the membranes at 150 L/h and heated to a temperature of 130 °C. A feed temperature of 130 °C was selected based on the thermal stability of the HybSi^®^ AR membranes and the goal of achieving high water flux across all tested solvent–water systems. Operating at elevated temperatures significantly enhances permeation flux, thereby reducing the required membrane area and improving the overall economic feasibility of the process. Although this temperature exceeds the atmospheric boiling point of the solvents, the pervaporation system was operated in a closed, sealed configuration without active pressure control on the feed side. As a result, pressure increased naturally during heating, guided by vapor–liquid equilibrium (VLE) behavior, which ensured that the feed remained in a subcooled liquid state throughout the experiment. No boiling or vapor formation was observed under any conditions.

This self-pressurization strategy allowed for safe high-temperature operation and reflects realistic process conditions where distillation column overheads, typically available at elevated temperatures, can be used as feed for the pervaporation unit without the need for additional heating. This direct thermal integration is made possible by the robust temperature tolerance of HybSi^®^ AR membranes, offering a distinct advantage over polymeric membranes that are limited to lower temperature ranges. A vacuum pump was used to reduce the vacuum pressure in the permeate to 20 mbar and the permeate was condensed using a condenser set at a temperature of 1 °C and collected in the permeate vessel. Samples were drawn from the permeate and the retentate to monitor the pervaporation process at different time intervals and feed and retentate temperatures were logged. Samples were analyzed for mass, using a precision balance (Kern PNJ 3000-2M, Balingen, Germany), and water content, using coulometric Karl Fischer titration (Aquacounter AQ-300, Ibaraki, Japan) and volumetric Karl Fischer titration (Metrohm 870 KF Titrino plus, Herisau, Switzerland). The water and solvent fluxes were determined using the following equation:

(1) $$J_{x}=\frac{m_{x}}{A\cdot t}$$

where *J_x_* is the flux (in kg m^−2^ h^−1^) of component *x* (water or isopropanol), and *m_x_* represents the mass of the permeating species (typically water), collected during the experiment per unit of membrane area, *A* (in m^2^), and per unit of time, *t* (in h).

At the start of the pervaporation experiment, the system is unable to keep the temperature isothermal at 130 °C due to the high flux. In this case, water flux was extrapolated based on the difference in driving force determined at the experimental temperature and at 130 °C using the VLE relation data for each solvent-water system. The driving force in pervaporation is the partial pressure gradient across the membrane of the permeating component.

The membrane’s separation performance was further characterized using the water/solvent separation factor (*α*), calculated based on the molar concentration ratios in the permeate and feed using the following expression [47]:

(2) $$\alpha_{water/solvent}=\frac{\left(y_{water}/y_{solvent}\right)}{\left(x_{water}/x_{solvent}\right)}$$

where *x* and *y* are the respective mass fractions of water and solvent in the feed and permeate.

The full experimental results, including measured fluxes and calculated separation factors under the actual operating conditions, are presented and discussed in Section 3.1.

The obtained water flux at a feed temperature of 130 °C and the appropriate vacuum pressure was plotted as a function of water content in the feed. The experimental data were fitted and an average water flux for each solvent was calculated between the feed and retentate water concentration required for the modeling. For all solvents except ACN, the organic flux is the result of the defects present in the membrane layer. As it is not caused by a driving force across the membrane, the flux cannot be fitted to the experimental data. Therefore, the maximum organic flux found for the solvent dehydrations of IPA, THF, ACA, and NMP was taken as the average organic flux for modeling. ACN, being a smaller-sized molecule compared to the others, is able to permeate through the membrane. In this case, the experimentally obtained ACN flux was fitted, and an average organic flux was calculated between the feed and retentate water concentration and used for the modeling.

### 2.2. Process Modeling

All the benchmark and the pervaporation-based (hybrid and standalone) processes for all solvent-water systems were developed and analyzed through Aspen Plus V14 simulations [48]. It is important to clarify that only the single-stage pervaporation (PV) experiments were performed in the laboratory to measure membrane fluxes and water/solvent separation behavior. The pervaporation units embedded in these models were calibrated using the experimentally obtained flux and separation factor data. This ensured that the simulated processes accurately reflect the performance of the HybSi^®^ AR membranes under realistic operating conditions and provide credible comparative assessments in terms of energy demand, cost, and environmental impact. Minor organic fluxes were detected in all pervaporation experiments. These are attributed to acceptable imperfections in commercial-grade HybSi^®^ AR membranes and are consistent with the reported behavior of ceramic membranes under vacuum and elevated temperature. To ensure realistic modeling, these small solvent permeation values were incorporated into the Aspen Plus simulation by adjusting component split fractions. This approach enables alignment between simulation results and experimental outcomes, particularly for solvent purity and recycle loop analysis in hybrid systems. The non-random two-liquid (NRTL) property method was used to accurately predict the thermodynamic properties and phase equilibria [49]. Pervaporation module (includes membranes, housing, and seals/gaskets) was modeled using a customized separator block, where split fractions for water and organic components were calculated from experimental permeation data. These fluxes reflect the membrane’s water/solvent separation factor and were adjusted to match observed mass balances across all test cases. The distillation columns were simulated using the Rad-Frac model, while the other equipment such as heaters, coolers, and pumps, were simulated using standard components available in Aspen Plus. The optimum number of stages, feed stage, and reflux ratio to achieve the desired product composition were estimated either by manual iteration or by using the design specs option. For the chiller, energy and cost data from the literature were used in the technical and economic assessment. The pressure drop in all equipment was neglected due to its negligible effect on the overall energy consumption. More details on process modeling are given in our previous work [50].

#### 2.2.1. Azeotropic Distillation

**Figure 2:**
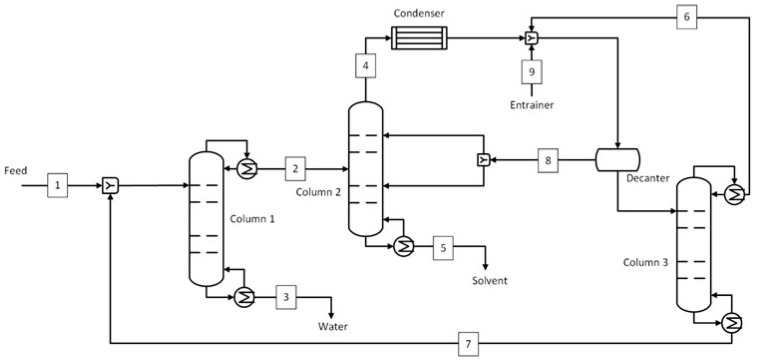
Azeotropic distillation process for IPA-H_2_O system (benchmark).

The azeotropic distillation using benzene as an entrainer was used as the benchmark for the IPA-H_2_O system. It was chosen based on literature studies that confirm it as the industry standard for IPA dehydration [15,51]. The process shown in Figure 2 consists of three distillation columns dedicated to water removal (column 1), breaking of azeotrope (column 2), and benzene recovery (column 3). The feed was sent to column 1, where most of the water is removed at the bottom (stream 3) at 99.8 wt.% purity while maintaining the top stream at a desired water concentration, 15 wt.% in this case. The top stream was sent to column 2, where the azeotrope was broken by using benzene as an entrainer. Here, the IPA is recovered from the bottom (stream 5) at the target purity of 99.5 wt.%. The top stream (stream 4), consisting of a mixture of IPA, benzene, and water, was condensed and sent to a decanter, where a benzene-rich stream and a water-rich stream were obtained. The benzene-rich stream (stream 8) was recycled to column 2, whereas the water-rich stream was sent for benzene recovery in column 3. The benzene was recovered from the top (stream 6) while the bottom stream, similar in composition to the initial feed mixture, was recycled to column 1. A small amount of benzene, which was lost with water (stream 3) and IPA (stream 5), was made up from stream 9. The stream condition at important locations, as shown in Figure 2, is shown in Table S1.

#### 2.2.2. Extractive Distillation

**Figure 3:**
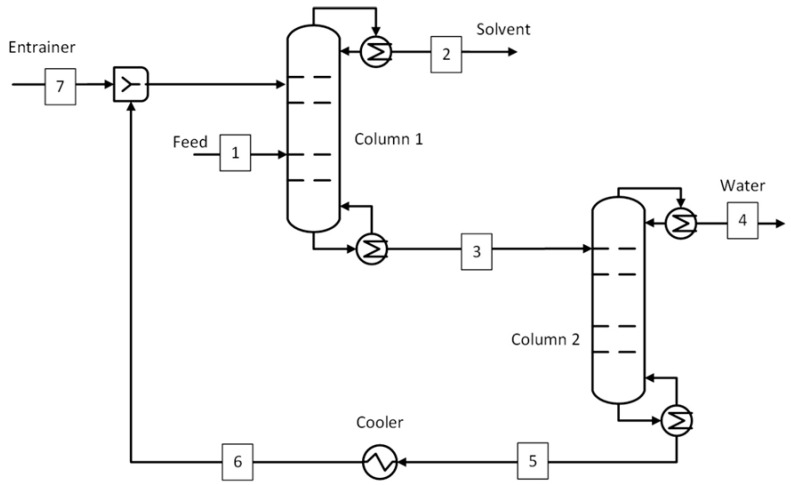
Extractive distillation process for ACN-H_2_O and ACA-H_2_O systems (benchmark).

The extractive distillation process is used as the benchmark for the ACN-H_2_O and ACA-H_2_O systems. Several literature studies recommend extractive distillation for the ACN-H_2_O system but with several different entrainers [43,52,53,54]. For the current study, ethylene glycol was chosen as the entrainer due to its low vapor pressure, low viscosity, and low toxicity. The amount of ethylene glycol required to eliminate the azeotropic point was also expected to be lower than the other entrainers [43]. The acetic acid does not form an azeotrope with water but has a boiling point close to water (118 °C vs. 100 °C). Therefore, separation using a simple distillation column is not a suitable option. An extractive distillation process is recommended in the literature with several suitable entrainers [44,55]. In the current study, adiponitrile is chosen due to lower heating and cooling duty requirements compared to azeotropic distillation with isobutyl acetate as an entrainer and practically no entrainer loss [44]. The process flow diagram of the extractive distillation process is shown in Figure 3. The feed was supplied to the extractive distillation column, where the solvent is separated with the help of an entrainer by altering the relative volatility of the components. The separated solvent was obtained from the top (stream 2) at a target purity of 99.5 wt.% while the bottom stream, consisting of a mixture of water and entrainer, was sent to the recovery column. Here, the entrainer was separated from water (stream 4) and recycled to the extractive distillation column after cooling (stream 6). The entrainer lost in the solvent and water streams was made up (stream 7) by mixing it with the recycled entrainer. The stream conditions at key plant locations shown in Figure 3 are shown in Table S5 and Table S13 for ACN-H_2_O and ACA-H_2_O systems, respectively.

#### 2.2.3. Pressure-Swing Distillation

**Figure 4:**
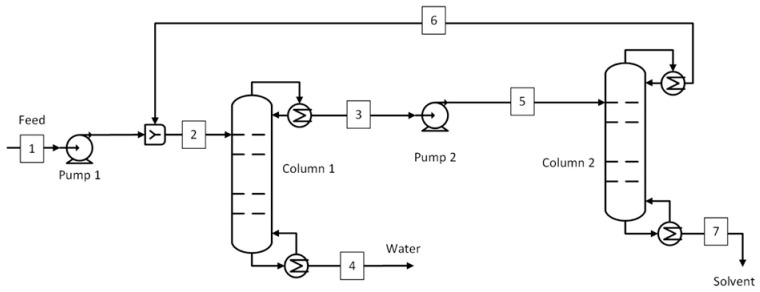
Pressure-swing distillation process for the THF-H_2_O system (benchmark).

The pressure-swing [45,56] and extractive distillation [56,57,58] processes are reported in the literature to separate the THF-H_2_O azeotropic mixture. The pressure-swing distillation shown in Figure 4 was chosen as the benchmark for the THF-H_2_O system due to two main reasons. The azeotropic point of the THF-H_2_O mixture can be changed by changing pressures, while the absence of an entrainer makes it relatively more environmentally friendly. The current process model is based on the model developed by Lee et al. [45], with the only difference being THF product purity. The feed mixture (stream 1) with a 1:1 ratio of THF and water was pressurized to 2.5 bar before feeding to the low-pressure column, which is also called the water removal column (column 1). This column operates at 1.35 bar with a column pressure drop of 0.3 bar. The bottom stream (stream 4) consists of water at 99.5 wt.% purity at 1.35 bar, while the top stream (stream 3) is a THF-rich stream at 1.05 bar. This stream is then pumped to 8 bar before feeding it to the THF recovery column which is also called a high-pressure column (column 2). This column operates at 7.3 bar with a column pressure drop of 0.3 bar. The THF was recovered from the bottom stream (stream 7) at 99.5 wt.% purity while the top stream at 7 bar was recycled to the initial feed. The stream conditions at important plant locations are given in Table S9.

#### 2.2.4. Vacuum Distillation

**Figure 5:**
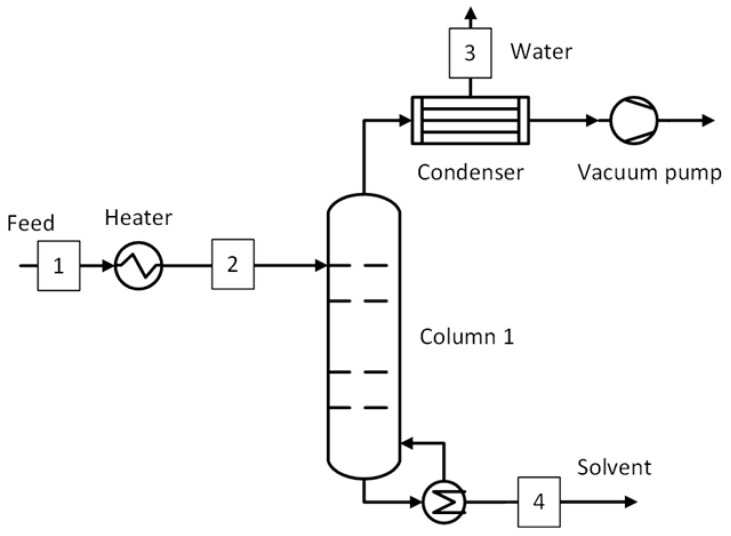
Vacuum distillation for NMP-H_2_O system (benchmark).

A vacuum distillation process shown in Figure 5 is considered a benchmark for the NMP-H_2_O system. Distillation is the most common method to separate NMP from water at atmospheric pressure [46,59]. However, it is an energy-intensive process due to the high boiling point of NMP, which is 202 °C. The extractive distillation, though it consumes relatively less energy, was not considered for benchmarking since it involves an entrainer that would need to be recovered, resulting in additional capital and operating expenses. Moreover, there is insufficient data available in the literature on extractive distillation for NMP separation [46]. Therefore, a vacuum distillation process with a single column based on Nurjanah et al. [46] was considered as a benchmark. The feed stream consisting of an equal mixture of NMP and water was heated to 80 °C and fed to column 1. A vacuum of 0.1 bar was applied at the top of the column and the condenser. The water at 99.5 wt.% purity was obtained at the top (stream 3) at 46 °C, while NMP at the same target purity was obtained at the bottom (stream 4) at 112 °C. The stream conditions at key plant locations shown in Figure 5 are given in Table S17.

The top stream consists of all condensable components, which are assumed to be condensed before the vacuum pump. Therefore, the power consumption of the vacuum pump was estimated only for the air leaked into the system using the methodology defined by Seider et al. [60]. The air leakage rate was estimated using Equation (3), where *W* is the air leakage rate in lb/hr, *P* is the operating pressure in torr, and *V* is the equipment volume in ft^3^. The column length and diameter were used to calculate the equipment volume. The brake power (*B_kw_*) was calculated using Equations (4) and (5), assuming a reciprocating vacuum pump where *SF* is the size factor. Though these equations are valid for *SF* = 1.0–25, it was assumed that these are also valid for *SF* lower than 1. For dissipating heat generated due to compression in the vacuum pump, cooling water was also required, which was estimated using a separate Aspen model, and it amounted to 7.3 kg/kg of air.

(3) $$W=5+\left\{0.0298+0.03088\left[ln\left(P\right)\right]-0.0005733{\left[ln\left(P\right)\right]}^{2}\right\}V^{0.66}$$

(4) $$B_{kw}=3.974*{\left(SF\right)}^{0.963}$$

(5) $$SF=\frac{W}{P}$$

#### 2.2.5. Hybrid and Standalone Pervaporation Processes

The two hybrid processes, distillation-pervaporation (D-PV) and distillation-pervaporation-distillation (D-PV-D), and the standalone pervaporation (PV) process have the same process of pervaporation. The feed temperature to the pervaporation module (pervaporation temperature) was raised to 130 °C using a heater and the vacuum pressure on the permeate side was kept at 20 mbar using a vacuum pump. The feed pressure was adjusted based on the VLE data to maintain the feed in a liquid phase for all solvent systems. The membrane modules were sized based on the permeate flux and purity data that were obtained from experiments. Depending on the required membrane area, the pervaporation may be performed in multiple modules, resulting in evaporative cooling and decreased pervaporation efficiency. Therefore, an interstage heater was also considered to reheat the feed to pervaporation temperature before every module. The heat required in the interstage heater was assumed equal to the heat of vaporization of all the permeate water. The saturation temperature of the permeate was different for different solvent systems. Therefore, for consistent comparison, a chiller [61] was used for permeate condensation in all cases. The cooling duty required in the condenser was obtained from the process models, while the power consumption was calculated using the COP of the chiller (3.4) [61]. The cooling of product streams was not considered in the current study.

**Figure 6:**
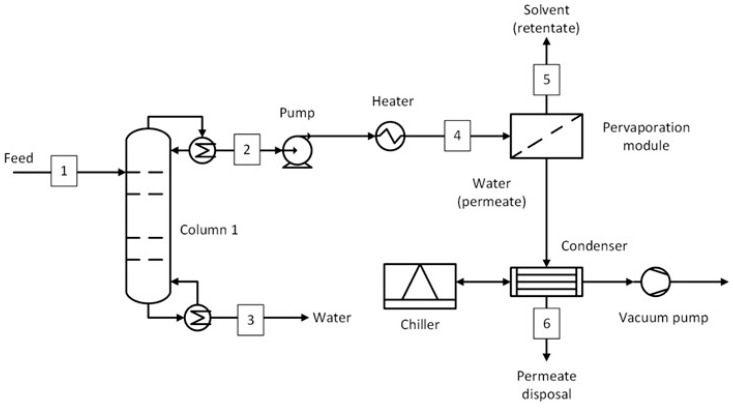
Hybrid distillation-pervaporation (D-PV) process.

The process flow diagram of the D-PV process is shown in Figure 6. A distillation column was used to remove water from the bottom (stream 3) and bring the top composition (stream 2) near the azeotropic point for IPA, ACN, and THF solvents. The concentration of water in the bottom stream was kept the same at 99.8 wt.% for IPA and ACN solvents, except for THF, where it was 99.99 wt.% due to limitations from the mass balance. The retentate stream (stream 5) consists of the recovered solvents with a targeted purity of 99.5 wt.%. The process is the same for ACA and NMP solvents except that the bottom stream of the distillation column removes a significant amount of solvent at the target purity. The rest of the solvent was recovered as retentate from the pervaporation module, also at the target purity. Since almost all the water is removed in the permeate, the membrane area and the condenser cooling duty required were relatively larger. The stream conditions at the plant key locations for the five solvent systems are given in Tables S2, S6, S10, S14 and S18.

**Figure 7:**
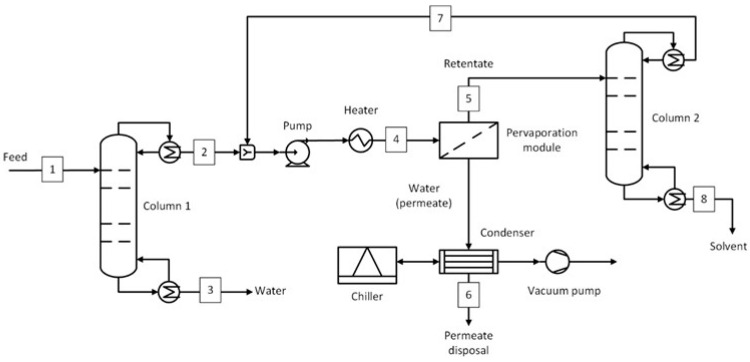
Hybrid distillation-pervaporation-distillation (D-PV-D) process.

The process flow diagram of the D-PV-D process is shown in Figure 7. This process has a second distillation column downstream of the pervaporation module which was used only to break the azeotrope (in case of azeotrope-forming solvents) or remove a significant amount of water (in case of non-azeotrope solvents). The retentate stream (stream 5) was sent to the second distillation column. This stream consisted of 5 wt.% water for all solvent systems except THF-H_2_O which had 2 wt.% water. The different specifications for the THF-H_2_O system were due to a lower azeotropic composition. The solvent was recovered from the bottom of the second column, and the top stream (stream 7) was recycled as feed to the pervaporation module. The stream conditions at the process important locations for the five solvent systems are given in Tables S3, S7, S11, S15 and S19.

**Figure 8:**
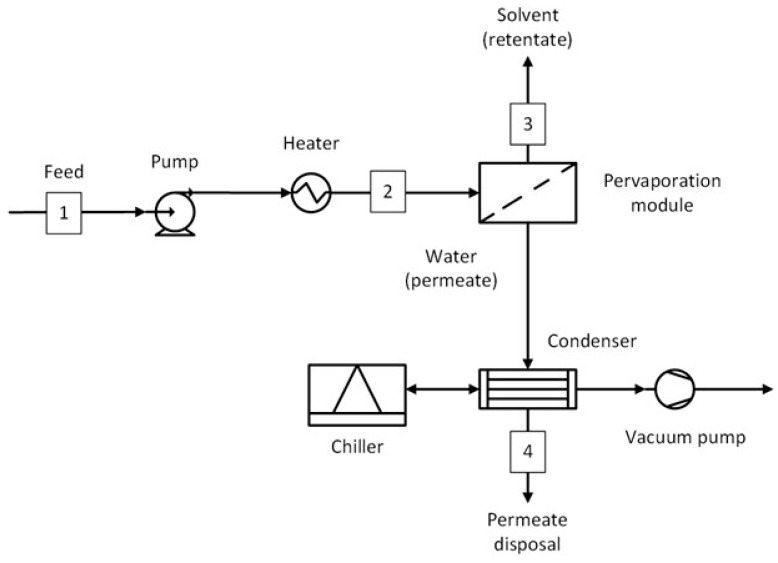
Standalone pervaporation (PV) process.

The process flow diagram of the standalone PV system is shown in Figure 8. In this process, the feed stream was directly fed to the pervaporation module at 130 °C. The rest of the downstream process is the same as for the hybrid processes. The conditions of important streams for all five solvent systems are shown in Tables S4, S8, S12, S16 and S20.

### 2.3. Economic Assessment Methodology

This section presents the methodology used to estimate the capital and operating costs. For the capital costs, all the equipment shown in Figure 2, Figure 3, Figure 4, Figure 5, Figure 6 and Figure 7 were considered, including the interstage heater. For the operating costs, all fixed costs, such as insurance, maintenance, and labor, and variable costs, such as utilities, membrane replacement, and waste disposal, were considered. More details on the economic assessment are given in our previous work [50].

#### 2.3.1. Capital Costs

The capital cost estimation methodology by Sinnott and Towler [62] was used in this study. The purchase costs (PC) were obtained from the Aspen process economic analyzer for all equipment except the pervaporation module and chiller. The price of the pervaporation module (membrane and pressure housing) used in this study cannot be disclosed due to confidentiality agreements. A chiller from Samsung costing €17,700 for a cooling capacity of 42 kW was considered [61,63]. The equipment cost was scaled using an appropriate scaling exponent in Equation (6) when required. An installation factor (IF) specific to each equipment was multiplied by the equipment PC to calculate the total cost of installed equipment (TIC). Additional multipliers for offsite (OS), design and engineering (D&E), and contingency (X) costs were used to arrive at the total plant cost (TPC). For comparison among the different cases, the annualized capital costs were calculated using the weighted average cost of capital (WACC) which in turn is dependent on the discount rate, equity to debt ratio, tax, and interest rates. All the data and assumptions used in capital cost estimation are presented in Table 2. The equipment lifetime, installation factors, scaling exponents and the operating personnel required are listed in Table 3.

(6) $$Cost\;of\;equipment\;A=\left(Cost\;of\;equipment\;B\right)\times {\left(\frac{Capacity\;A}{Capacity\;B}\right)}^{Exponent}$$

#### 2.3.2. Operating Costs

The fixed operating costs, such as insurance and maintenance, are calculated as a percentage of TPC. The required operating labor was estimated by summing the requirements of each equipment. For all cases, two operating personnel were required with an average salary of €60,000/yr per person, which is typical for an experienced plant operator in Belgium [70]. The variable operating costs include the cost of energy, membrane replacement, and waste disposal in Belgium. The required steam saturation temperature was different for different solvent systems. The steam price based on the natural gas price was estimated for three different pressures (8.75 bar, 39.5 bar, and 85 bar) using an online application [71]. The inlet and outlet temperatures of the cooling water required in the condensers of the distillation columns were 15 °C and 25 °C, respectively. The separated water was contaminated by the organic fraction in both benchmark and pervaporation-based processes, needing an appropriate treatment before releasing into public drains. A purification and disposal cost of €3.2/m^3^ was considered which was in line with wastewater treatment costs in Belgium [72,73]. The other variable operating cost is membrane replacement, which is performed every 5 years. Assuming a longer module lifespan, only the membrane cost was considered in addition to the installation cost. The assumptions and data used for the operating cost estimation are presented in Table 4. The price of entrainers used in the azeotropic and extractive distillation benchmark processes is also listed in the table.

### 2.4. Environmental Analysis

The annual CO_2_ emissions resulting from the utilities and chemicals consumption were estimated using their standard CO_2_ emission intensities shown in Table 5. CO_2_ was chosen as a representative emission due to its direct correlation with energy consumption and widespread use in environmental assessment. Other emissions such as SO_2_ and NO_x_ were not considered, as their impact was deemed negligible based on clean electricity use assumptions. The annual CO_2_ emissions arethe sum product of the quantity of utilities/chemicals and their corresponding emission intensities. The emission intensity of electricity chosen was for the Belgian electricity mix in 2023 [80]. The use of electricity was mainly by the pumps, chiller, and vacuum pump. The steam was considered to be produced in a natural gas-fired boiler. The same emission intensity was used for steam at different pressures due to slight differences in energy consumption. Steam was used in preheating the feed and in the reboilers of the distillation columns. The emission intensity of cooling water is very small due to the assumption that it is recycled. The entrainers used in the azeotropic and extractive distillation processes were lost through the product streams. Depending upon the makeup amount, their contribution to the overall CO_2_ emissions may be significant.

### 2.5. Performance Metrics

The technical performance of the pervaporation-based processes was compared with the benchmark processes using the recovery efficiency (*η_rec_*) and energy intensity (*ε*) shown in Equations (7) and (8). The recovery efficiency is the ratio of the amount of solvent recovered (*ṁ_SOL_outlet_*) to the amount of solvent in feed (*ṁ_SOL_feed_*). This parameter indicates the amount of solvent lost with the permeate, which may have a significant effect on the separation costs. The solvent loss also affects the energy intensity, which is the amount of energy required to recover a unit tonne of solvent. Here, the total energy is the sum of electricity (*Ẇ_el_*) and enthalpy of steam (ṁ_st_*Δ*h*).

(7) $$\eta_{rec}=\frac{{\dot{m}}_{SOL\_outlet}\;}{{\dot{m}}_{SOL\_feed}}$$

(8) $$\varepsilon =\frac{{\dot{W}}_{el}+{\dot{m}}_{st}*\Delta h}{{\dot{m}}_{SOL\_outlet}}$$

The economic performance was compared by using the levelized cost of separation (LCOS) shown in Equation (9). It is the ratio of annualized capital (*C_capex_*) and operating (*C_opex_*) costs to the amount of solvent recovered annually. The environmental performance was compared by using the emission intensity (*E*) shown in Equation (10). It is the ratio of annual CO_2_ emissions described in Section 2.4 to the amount of solvent recovered annually.

(9) $$LCOS=\frac{C_{capex}+C_{opex}}{{\dot{m}}_{SOL\_outlet}}$$

(10) $$E=\frac{{\dot{W}}_{el}*E_{el}+{\dot{m}}_{st}*E_{st}+{\dot{m}}_{CW}*E_{CW}+{\dot{m}}_{B}*E_{B}}{{\dot{m}}_{SOL\_outlet}}$$

A single parameter combining the *LCOS* and emission intensity was defined to compare the pervaporation-based processes with their corresponding benchmark process. It is named as the COPCO index, which stands for COst savings/expenditure of the Pervaporation systems per unit tonne of CO_2_ saved/emitted and calculated using Equation (11). The numerator is the difference between the LCOS while the denominator is the difference between the emission intensity of benchmark (*ben*) and pervaporation (*per*) processes. The resulting number (X) is denoted by preceding and succeeding signs (−X− or +X− or −X+ or +X+). The preceding positive and negative signs indicate that the pervaporation process either saves or increases the cost, respectively. The succeeding positive and negative signs indicate that the pervaporation process either saves or emits CO_2_, respectively. Ideally, both cost and emissions savings are desired (+X+) but this may be different for different solvent systems.

(11) $$COPCO=\frac{{LCOS}_{ben}-{LCOS}_{per}}{E_{ben}-E_{per}}$$

## 3. Results and Discussion

The results from the pervaporation experiments using HybSi^®^ AR membranes and the economic and environmental assessments are presented in this section. The experimental results, including the average permeate flux and permeate quality and their implications on the membrane area, are discussed. The technical performance of the pervaporation-based processes in terms of utility consumption, recovery efficiency, and energy intensity is discussed in comparison with the benchmark processes. Subsequently, the results of the economic and environmental performance in terms of the *LCOS*, emission intensity, and *COPCO* index are also discussed. Lastly, important results from the sensitivity analysis are also discussed here for some solvent systems, and the remaining results are given in the Supplementary Material. The results for IPA presented in this study are reproduced from our earlier publication for consistency and comparative analysis [50].

### 3.1. Experimental Pervaporation Results

**Figure 9:**
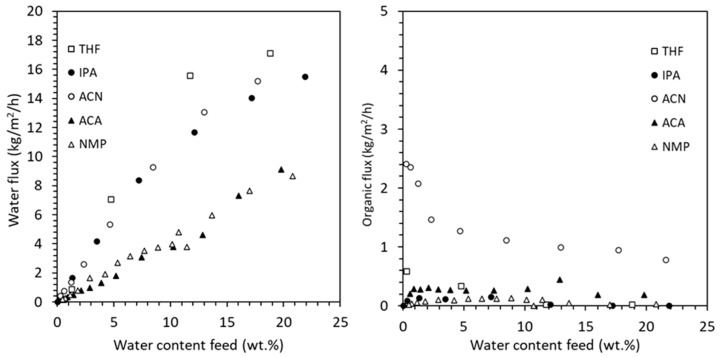
Water and organic flux for five solvent-water systems as a function of feed water content.

Figure 9 presents the measured water flux (left) and organic flux (right) as a function of water content in the feed for all five solvent–water systems tested using HybSi^®^ AR membranes. As expected, water flux increases with feed water content due to the enhanced driving force, with THF and ACN exhibiting the highest water fluxes. In contrast, organic solvent fluxes remain low across all systems, confirming a high water/solvent separation factor. Slightly elevated organic flux was observed for ACN, likely due to its low viscosity and small molecular size.

While vapor pressure and molecular size are important determinants of pervaporation flux, the observed differences between solvents also reflect the influence of solvent-specific properties. In HybSi^®^ AR membranes, separation primarily follows a molecular sieving mechanism. However, the effective flux is also impacted by factors such as membrane–solvent interactions, solvent viscosity, and polarity. For instance, acetonitrile (ACN) exhibited higher flux than NMP, which can be attributed not only to its smaller molecular size but also to its lower viscosity and weaker interaction with the membrane material. In contrast, NMP’s higher viscosity and stronger hydrogen bonding likely reduced transport rates, even under similar operating conditions. This indicates that a combination of steric, thermodynamic, and transport-related effects governs permeation performance.

The corresponding separation factors (α), summarized in Table 6, ranged from 85 (ACN) to 977 (IPA), further confirming the membrane’s strong preference for water transport. It is also important to note that, at the start of some experiments, the system was unable to maintain isothermal operation at 130 °C due to high initial flux. In these cases, the measured fluxes at slightly lower temperatures were extrapolated to 130 °C using vapor–liquid equilibrium (VLE) data to correct for differences in driving force. This ensures consistency in the performance comparison and provides input for accurate process simulation.

The average water and solvent fluxes, along with the permeate quality (or selectivity) obtained from experiments, are presented in Table 7. For the hybrid processes (D-PV and D-PV-D), the inlet water concentration differs for every solvent. This concentration was fed to the pervaporation module and was obtained after the first distillation column. For the solvents IPA, ACN, and THF, the inlet water concentration was kept near their azeotropic compositions. For the solvents ACA and NMP, which do not form azeotropes, the inlet water concentration was adjusted to ensure that most of the solvent was recovered from the bottom at target purity while maintaining the mass balance in the column. For the standalone PV process, the inlet water concentration was the same as the initial feed (50 wt.%). The outlet water concentration was kept the same for each process in the solvent systems. The processes D-PV and PV had 0.5 wt.% as the outlet water concentration, which is equal to the target purity. In the D-PV-D process, an outlet water concentration of 5 wt.% was selected for all solvents to break the azeotrope, except for THF, which required a concentration of 2 wt.% due to its lower azeotropic composition.

The general trend is that by increasing the inlet water concentration while keeping the outlet water concentration constant, the average water flux also increases. The driving force in pervaporation is a chemical potential gradient, which is largely determined by the concentration of the permeating component (water in this case) in the feed. As the water concentration decreases in the feed, the chemical potential difference between the feed and permeate side diminishes, leading to lower water flux through the membrane. Similarly, when the inlet water concentration was kept constant and the outlet water concentration was increased, the average water flux obtained was higher. This is due to avoiding lower flux values at lower concentrations (such as at 0.5 wt.%) while time-averaging the experimental data. The standalone PV process can be used to compare the average water flux for all solvents since the inlet and outlet water concentrations are the same. The average water flux is in the following order from highest to lowest values: ACN > IPA > THF > ACA > NMP. The required membrane area is not solely determined by the average water flux, but it also depends on the water content and flow rate of the feed stream. The solvent flux remains relatively low across all cases, indicating a high water/solvent separation factor and effective separation. The ACN solvent has the highest flux, while the NMP solvent has the lowest flux, which is also reflected in the permeate quality. Among other reasons, ACN is a small molecule and has a higher vapor pressure than NMP, which drives a greater flux through the membrane during the pervaporation process.

### 3.2. Technical Performance

The technical performance in terms of required membrane area, recovery efficiency, energy and utility consumption, and energy intensity for all solvent systems is shown in Table 8. The membrane area was calculated based on the average flux and permeate flow rates obtained from the experiments. The standard module generally has 3 m^2^ of membrane area, but a design of 10 m^2^ is also available in the market, and smaller modules, depending upon the requirement, can also be designed. The recovery efficiency allows for quantification of the solvent loss in the permeate stream and assists in comparison between the benchmark and pervaporation-based processes. The cooling water shown here represents the cooling duty required for a particular process and thus is not consumed. It is assumed that it is recycled entirely except for some evaporative losses. This metric is particularly important in selecting the separation process suitable for water-scarce locations. The energy consumption in terms of electricity and steam allows for comparison and identification of the primary energy source of all the processes. This will help in choosing the process that primarily relies on the preferred energy type (electricity or heat). It will also help in accurately sizing the equipment associated with energy supply, such as an on-site boiler for steam generation. If the energy type is not the main concern, then the energy intensity, which includes both electricity and heat, could be used for overall comparison.

For IPA dehydration, the recovery efficiencies remain almost the same for all four cases investigated, with the highest recovery efficiency of 99.8% obtained by the D-PV-D case. Further, the D-PV-D case also requires the smallest membrane area due to the use of pervaporation to only break the azeotrope. The largest membrane area was required in standalone PV, where all the feed water is removed on the permeate side, resulting in the largest electricity consumption (by vacuum pump) compared to other cases. When it comes to steam requirements, the benchmark azeotropic distillation emerged as the most energy-consuming option, which is also reflected in its energy intensity (2.0 MWh/t-IPA). The lowest steam was required in the D-PV process, resulting in an energy intensity of only 0.9 MWh/t-IPA, emerging as the most energy-efficient option. The AD case has the largest cooling water requirement, mainly in the condensers of the three distillation columns. The cooling water requirement follows the number of distillation columns involved. The higher the number of distillation columns, the higher the cooling water requirement. While AD and D-PV-D offered higher recovery efficiencies, their higher utility consumption made them less favorable. The standalone PV with no distillation columns requires the largest membrane area and has the lowest cooling water requirement and relatively lower energy intensity, presenting a balanced approach.

The recovery efficiencies are variable among the four cases investigated for ACN dehydration. This variation stems from the fact that the solvent loss observed in the permeate during the experiments was significantly higher, as shown in Table 7. Though the benchmark ED case has the highest recovery efficiency (99.5%), its high energy intensity, steam, and cooling water consumption make it highly unattractive. The hybrid D-PV option has the lowest energy intensity (1.0 MWh/t-ACN) but suffers from relatively lower recovery efficiency and considerable cooling water consumption. The hybrid D-PV-D option with the lowest membrane area requirement offers moderate recovery efficiency and energy intensity. However, the cooling water requirement was significantly larger, making it unattractive for water-scarce locations. The standalone PV case has the lowest cooling water requirement, making it suitable for water-scarce locations. Further, with lower energy intensity (1.1 MWh/t-ACN), this can be an attractive option if the solvent losses can be ignored.

For THF dehydration, not much variation was observed in the recovery efficiencies, with the highest being 99.8%, achieved by the hybrid D-PV-D case. The membrane area requirement observed was also higher than that required in the IPA and ACN solvent systems due to relatively lower permeate fluxes. The benchmark PSD and the standalone PV have similar energy intensities (1.1 vs. 1.0 MWh/t-THF), but the significant difference in the cooling water consumption makes the standalone PV a highly attractive option between the two. The hybrid D-PV and D-PV-D cases also show similar energy intensities (0.6 vs. 0.7 MWh/t-THF), but with lower cooling water requirements, the D-PV case emerged as the best option for THF dehydration.

Since ACA does not form an azeotrope and has a higher boiling point than water, it was recovered from two separate streams (column bottom and retentate streams). Consequently, a high inlet water concentration to the pervaporation module was observed, resulting in a higher membrane area requirement. The benchmark ED process achieved a complete solvent recovery and had moderate energy intensity (1.5 MWh/t-ACA) but suffered from significant cooling water consumption, making it unsuitable for water-scarce locations. The hybrid D-PV and D-PV-D processes have moderate recovery efficiencies, the highest energy intensities, and cooling water consumption, making them highly unattractive. The high steam requirement is due to the thermal energy required in the first distillation column, feed, and interstage heating of almost all the water. The standalone PV option with the lowest energy intensity (1.0 MWh/t-ACA) and cooling water consumption (483 m^3^/yr) emerged as the most suitable option for ACA dehydration but suffers from a lower recovery efficiency (96.6%) and needs a significantly larger membrane area (58 m^2^).

Like ACA, NMP also does not form an azeotrope with water, and it is recovered from two separate streams (column bottom and retentate streams). The benchmark VP process seems to be the best option with the lowest energy intensity (0.8 MWh/t-NMP), moderate recovery efficiency (99.5%), and cooling water requirement. The standalone PV emerged as the second-best option with an energy intensity of 1.0 MWh/t-NMP and a recovery efficiency of 98.9%. It also has the lowest cooling water requirement but needs a very large membrane area (68 m^2^) due to very low permeate flux (see Table 7), which may have a large influence on the costs. Despite achieving 100% recovery efficiencies, the hybrid D-PV and D-PV-D processes proved to be highly unattractive due to larger energy intensities and cooling water requirements.

### 3.3. Economic Performance

**Figure 10:**
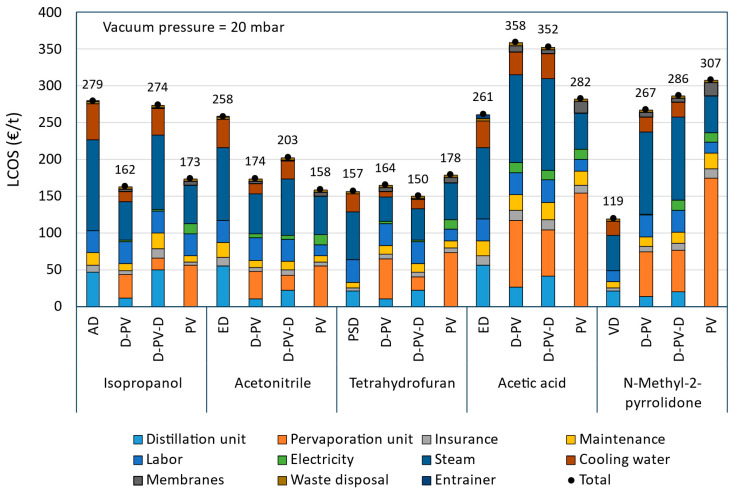
Breakdown of the levelized cost of separation.

The annualized capital and operating costs for all the cases investigated for five solvent systems are given in Table 9. The reduction in the LCOS with respect to their respective benchmark process is also given in the table, while the LCOS breakdown is presented in Figure 10. In the figure, the capital cost is divided into the distillation and the pervaporation units and is based on the economic data shown in Tables S21–S40. Specifically, the capital cost of the columns used is based on the design data shown in Tables S41–S45 of the Supplementary Information. The capital cost breakdown, including the contribution from all the equipment, is shown in Figures S2–S6, where the *IS heater* refers to the interstage heater and *pervap* consists of only the initial membrane costs, while the replacements are included in the operating costs.

The AD process serves as the benchmark for comparison among IPA dehydration processes. It has a high operating cost due to significantly high steam and cooling water consumption, as shown in Figure 10, resulting in the highest LCOS. The D-PV process shows some reduction in capital cost but a significant reduction in operating cost compared to AD, leading to a 42% reduction in the LCOS. This makes it a cost-effective alternative. The D-PV-D process has the highest capital cost due to the involvement of two distillation columns and consequently shows a high operating cost due to higher steam and cooling water demand, resulting in an LCOS close to that of AD, with only a 2% reduction. This indicates that the additional distillation step may not be cost-effective. The PV process has a moderate capital cost and the lowest operating cost among the processes compared, resulting in a 38% reduction in the LCOS. This makes it a competitive option in terms of cost-efficiency.

For ACN dehydration, the benchmark ED process has the highest capital and operating costs due to the involvement of two distillation columns that require a large amount of steam and cooling water, consequently leading to a very high LCOS. The D-PV process shows a considerable reduction in capital cost compared to ED due to the replacement of the second distillation column with the pervaporation module. It also shows a significant reduction in operating costs due to a reduction in utilities consumption, resulting in a 33% reduction in the LCOS. The hybrid D-PV-D process, though involving two distillation columns, has the lowest capital cost due to smaller-sized columns and a pervaporation module. However, it still requires a moderate operating cost, which leads to a 21% reduction in LCOS compared to ED. The standalone PV process shows a moderate capital cost and the lowest operating cost among the compared processes, resulting in a 39% reduction in the LCOS. This makes it the most cost-effective option for ACN dehydration.

The benchmark PSD process for THF dehydration has the lowest capital cost due to smaller-sized columns but has the highest operating cost due to high utility consumption among the four cases investigated. The operating costs are also lower than those observed in benchmarks AD and ED of IPA and ACN solvent systems, respectively. This is due to the absence of an entrainer that is necessary in AD and ED processes, which requires additional steam and cooling water in the columns. The D-PV process shows a significant reduction in the operating cost compared to PSD. However, it has the highest capital cost mainly due to the contribution from the pervaporation module. This results in a slightly higher LCOS (€164/t-THF), representing a 5% increase compared to PSD. This increase can be easily overcome at a lower membrane price or higher permeate flux, which will be discussed in Section 3.4 on sensitivity analysis. The hybrid D-PV-D process with moderate capital and operating costs emerged as the most cost-effective option with a 4% reduction in the LCOS compared to PSD. The potential for further reducing the LCOS by using cheaper membranes or increasing permeate flux is limited, as these factors contribute only modestly to the overall LCOS. The standalone PV process is the least cost-effective option as it shows the highest capital and moderate operating costs, leading to a 14% increase in the LCOS compared to PSD. Since the capital cost is mainly due to the pervaporation module, using cheaper membranes or increasing permeate flux has the potential to reduce the LCOS.

For ACA dehydration, the benchmark ED process showed the lowest capital and moderate operating costs, leading to an LCOS of €261/t-ACA. The hybrid processes D-PV and D-PV-D showed significantly higher capital and operating costs, resulting in a significant increase in LCOS (36% and 38%) compared to the ED process. Figure 10 shows that the pervaporation module’s capital cost and the utility consumption (steam and cooling water) of the operating costs are the main contributing factors. The standalone PV process has the highest capital cost among the four cases investigated, and this is only due to the expensive pervaporation module. However, the significant reduction in the operating cost leads to the LCOS of €282/t-ACA, which is only a 9% increase compared to the benchmark ED process. Further reduction in capital cost is only possible if the membrane price drops or with enhanced permeate flux, which can be realized by increasing feed temperature. Waste heat could be utilized for this; otherwise, this will influence energy consumption and overall costs.

For NMP dehydration, the VD process serves as the baseline with the lowest costs. Though NMP has a high boiling point, the use of a vacuum reduces the energy requirement in the distillation column. The pervaporation-based processes (D-PV, D-PV-D, and PV), despite their potential benefits, result in significantly higher LCOS due to their high capital and operating costs, with standalone PV as the least interesting option. The pervaporation module to the capital cost and the steam consumption to the operating cost are the main contributing factors. It is clear from Figure 10 that the likelihood of a reduction in LCOS below the benchmark VD process appears to be low. More discussion on this is presented in Section 3.4 on sensitivity analysis.

### 3.4. Sensitivity Analysis

This section discusses the sensitivity of the LCOS to various technical and economic parameters. The parameters selected for sensitivity analysis include feed flow rate, permeate flux, membrane module price, membrane lifetime, steam price, and cooling water price. The results are presented in Figures S7–S11 of the Supplementary Information, with only the most significant findings discussed here. Firstly, the general effect of these parameters is discussed, followed by key observations from the results of each solvent system. The feed flow rate varied from 100 to 3500 kg/h, with 1000 kg/h as the base value for all solvent-water mixtures. Typically, as the feed flow rate increases, LCOS might decrease due to economies of scale, but this can vary depending on system specifics. This is due to equipment such as distillation columns, vacuum pumps, chillers, etc., that scale non-linearly with their capacity. The pervaporation module was assumed to be modular and therefore scales linearly, not contributing to the economies of scale effect. Beyond a particular feed flow rate, the effect of economies of scale diminishes and eventually becomes negligible. However, at lower feed flow rates, the impact of economies of scale becomes more significant, potentially making the benchmark process more attractive in terms of LCOS. However, at these flow rates, operating a distillation column can present challenges, such as heat losses due to a high surface area-to-volume ratio, as well as control and stability issues. Therefore, pervaporation-based systems may be the best options at low flow rates.

The permeate flux varied from 2 to 30 kg/h/m^2^, with different base values for pervaporation-based processes of different solvent systems. The flux is directly proportional to membrane area, which is reflected in capital and operating costs. Higher permeate flux usually leads to lower LCOS as more product is obtained per unit of membrane area. However, beyond a certain flux value, the cost savings from reducing the membrane area are minimal due to its smaller contribution to LCOS.

The membrane module price (including membranes, housing, and seals/gaskets) varied from 20% to 160%, with 100% as the base value. An increase in membrane price generally raises the LCOS, reflecting higher capital costs, especially in processes that require a larger membrane area. Similarly, longer membrane life typically reduces the LCOS, as the replacement frequency and associated costs are lower. The membrane lifetime varied from 1 to 10 years, with 5 years as the base value. Beyond a certain lifetime value, the effect on the LCOS diminishes due to less contribution from the membrane cost towards the LCOS. Even if the pervaporation-based process has a higher LCOS than the benchmark, a combination of the aforementioned parameters may make the pervaporation-based process more attractive. However, achieving an optimum combination for some solvent systems may be practically impossible. Due to the absence of membranes in benchmark cases, the membrane-related parameters (permeate flux, membrane price, and lifetime) have no role, and thus they are represented as dotted lines in figures indicating the benchmark LCOS.

The steam price varied from €0 to €35/t, with €35/t as the base value, while the cooling water price varied from €0 to €0.35/m^3^ with €0.32/m^3^ as the base value. Higher steam and cooling water prices increase the LCOS due to higher operational costs. Steam production was assumed to come from a gas-fired boiler, making it dependent on the highly variable natural gas (NG) market price. Consequently, processes that rely more on electricity and less on thermal energy are preferred. The processes with fewer or no distillation columns are less affected by steam and cooling water prices due to their lower consumption. In contrast, processes with two or more distillation columns use more steam and cooling water, making them more sensitive to price fluctuations.

For the IPA-water system, at a feed flow rate of around 100 kg/h, the benchmark AD process outperforms pervaporation-based cases. However, such low flow rates may not be practical and economically feasible due to increased heat losses and operational challenges. For a permeate flux below 10 kg/h/m^2^, the benchmark AD process shows a lower LCOS than the D-PV-D and PV processes. The D-PV process has a lower LCOS than the benchmark AD process at all flux values. When the membrane module price exceeds 120% of the base value, only the D-PV-D process shows slightly higher LCOS than the benchmark AD process. The D-PV and PV processes show lower LCOS at all considered membrane module prices. The D-PV-D and PV processes require membrane lifetimes above 3 and 1.5 years, respectively, to be economically better than the benchmark AD process. For the ACN-water system, permeate flux and membrane lifetime are key parameters. The permeate flux needs to be above 4, 7, and 10 kg/h/m^2^ for the D-PV, D-PV-D, and PV processes, respectively, to be economical. The membrane lifetime needs to be above 1 year for all pervaporation-based processes to be economical.

For the THF-water system, only the PV process requires the feed flow rate to be above 500 kg/h to outperform the benchmark PSD process. The permeate flux needs to be above 5, 10, and 25 kg/h/m^2^ for the D-PV, D-PV-D, and PV processes, respectively, to be economical. The membrane module price needs to be below 80%, 90%, and 140% of the base value for the PV, D-PV, and D-PV-D processes, respectively, to be economical. Similarly, the membrane lifetime needs to be above 4 years for all pervaporation-based processes to be more economical than the benchmark PSD process. Steam and cooling water prices are important only for the D-PV-D process, as it involves two distillation columns. For the ACA-water system, only the standalone PV process has the potential to outperform the benchmark ED process. The feed flow rate needs to be below 500 kg/h, the permeate flux above 10 kg/h/m^2^, the membrane module price below 90% of the base value, and the membrane lifetime above 6 years for it to be economical. For the NMP-water system, none of the pervaporation-based processes show lower LCOS than the benchmark VD process. However, a combination of suitable permeate flux, membrane module price, and membrane lifetime values could potentially result in lower LCOS.

### 3.5. Environmental Performance

Table 10 presents the annual CO_2_ emissions and emission intensities for four cases in each solvent system. The reduction in CO_2_ emissions is primarily attributed to decreased steam and cooling water consumption. For the IPA-water, ACN-water, and THF-water systems, all pervaporation-based cases demonstrate significant reductions in CO_2_ emissions compared to their respective benchmark processes. The standalone PV case is the most effective, followed by the D-PV case, with the D-PV-D case showing the least reduction in CO_2_ emissions. In the ACA-water system, the standalone PV case achieves an 82% reduction in CO_2_ emissions compared to the benchmark ED case. The D-PV and D-PV-D cases exhibit similar or increased CO_2_ emissions, respectively. For the NMP-water system, the standalone PV case results in a 65% reduction in CO_2_ emissions compared to the benchmark VD process. However, the D-PV and D-PV-D cases show significant increases in CO_2_ emissions due to higher energy consumption.

### 3.6. Discussion

**Figure 11:**
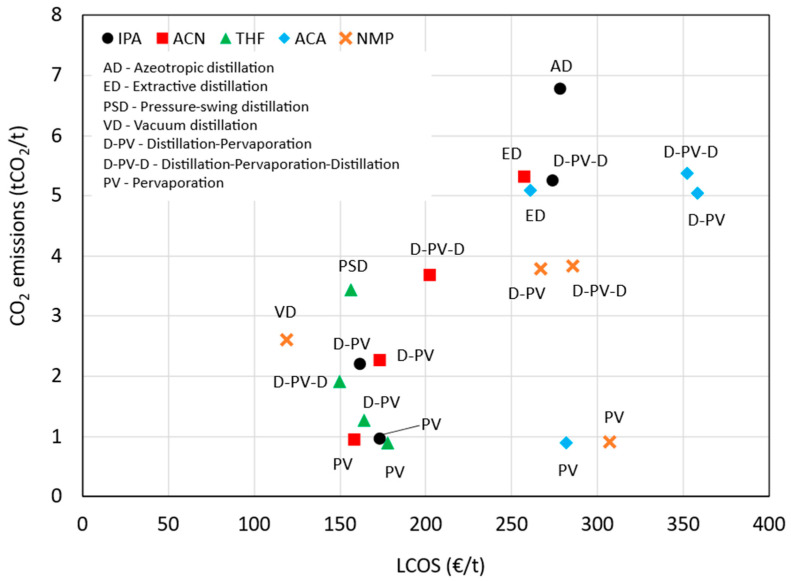
Mapping of the cost and CO_2_ emissions.

Figure 11 illustrates the comparison of the benchmark and pervaporation-based processes across all five solvent systems, highlighting their respective LCOS and CO_2_ emissions. The PV case generally has a lower LCOS compared to the benchmark and D-PV case, except for THF and ACA, where the LCOS are slightly higher. The D-PV case shows a significant cost reduction for IPA and ACN compared to the benchmark. The PV case consistently shows the lowest emissions across all solvent systems. The D-PV case also shows reduced emissions compared to the benchmark, but not as low as the PV case. The D-PV-D method shows variable LCOS relative to the benchmark and generally has higher emissions, except for THF. Overall, the PV method appears to be the most efficient in terms of both cost and emissions across all solvents, while the D-PV method also shows notable improvements over the benchmark.

**Figure 12:**
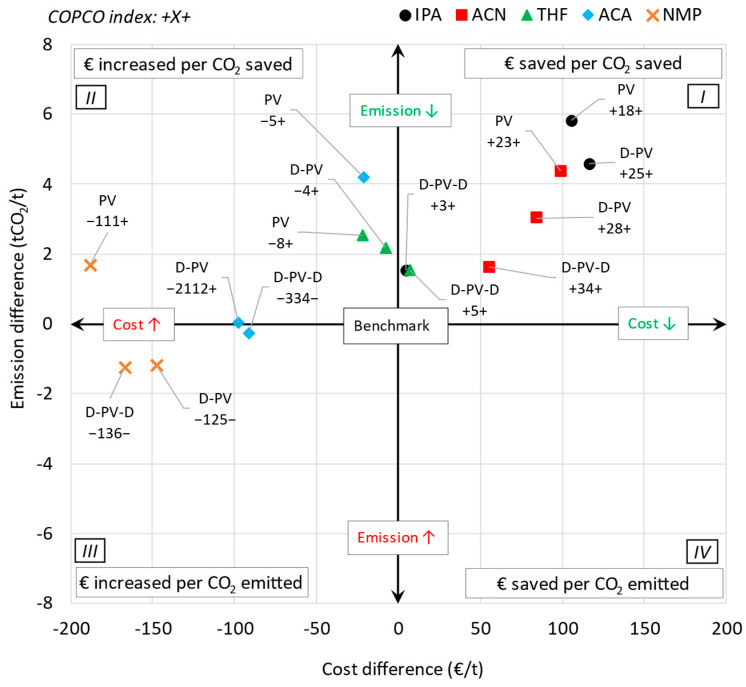
Mapping of the COPCO index (upward arrow—increase and downward arrow—decrease).

Figure 12 shows the mapping of the COPCO index of all the cases investigated. The *x*-axis represents the LCOS difference, while the *y*-axis represents the emission difference between a benchmark and a pervaporation-based process, with the origin representing the benchmark process. The labels represent the COPCO index, where the preceding sign indicates cost savings (+) or increases (−), and the succeeding sign indicates emissions reductions (+) or increases (−). Quadrant 1 (top right), with both LCOS and emission difference as positive, represents cases where both LCOS and emissions have decreased compared to the benchmark. Quadrant 2 (top left), with LCOS difference as negative and emission difference as positive, represents cases where LCOS has increased but emissions have decreased compared to the benchmark. Similarly, quadrant 3 (bottom left), with both LCOS and emission difference as negative, represents cases where both LCOS and emissions have increased compared to the benchmark. Lastly, quadrant 4 (bottom right), with LCOS difference as positive and emission difference as negative, represents cases where LCOS has decreased but emissions have increased compared to the benchmark.

The figure shows that for the IPA and ACN systems, all pervaporation-based cases fall in Quadrant 1. For the THF system, the D-PV and PV cases fall in Quadrant 2, but very close to Quadrant 1, while the D-PV-D case falls in Quadrant 1. Similarly, for the ACA system, the D-PV and PV cases fall in Quadrant 2, with the D-PV-D case falling in Quadrant 3, very close to Quadrant 2. For the NMP system, only the PV case falls in Quadrant 2, while the other cases fall in Quadrant 3. None of the investigated cases fall in Quadrant 4.

Recovering used solvents instead of purchasing virgin solvents is crucial for both cost savings and reducing CO_2_ emissions. Reclaiming solvents on-site can significantly lower the expenses associated with buying new solvents. The cost of purchasing virgin solvents can be quite high, and by recycling used solvents, companies can cut down on these recurring costs. Disposing of spent solvents can be expensive due to hazardous waste regulations. Recycling solvents reduces the volume of waste that needs to be managed and disposed of, leading to further cost savings. The process of recovering used solvents, especially dehydration, typically generates significantly lower CO_2_ emissions compared to producing virgin solvents from raw materials. Recycling solvents often requires less energy than producing new solvents, which further contributes to reducing the overall carbon footprint of the process. By recovering used solvents, industries can achieve substantial economic benefits while also contributing to environmental sustainability by lowering their carbon emissions. This approach supports the circular economy and helps in minimizing the environmental impact of industrial processes.

**Figure 13:**
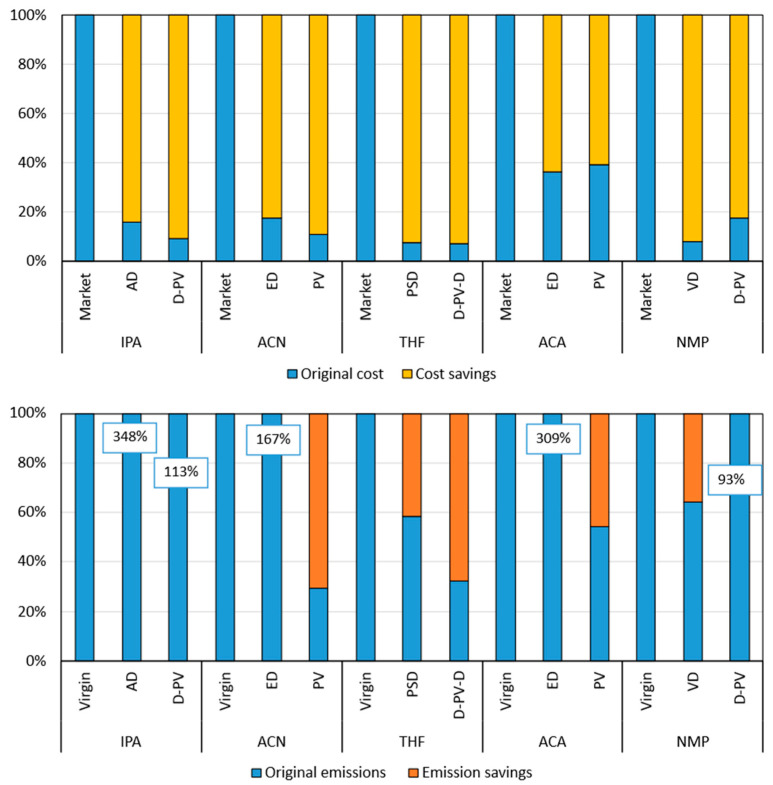
Cost and emission savings in recycling compared to market price and virgin solvent emissions.

Figure 13 shows the relative comparison of cost and CO_2_ emissions between the virgin solvent and the recovered (dehydration) solvent using the benchmark (traditional distillation processes) and the best pervaporation-based processes. The market price [82,83,84,85,86] and the emission intensity [87] of the virgin solvents are given in Table S47 of the Supplementary Information. The emission intensities were extracted from the ecoinvent database 3.9.1 as implemented in Simapro 7.3.3 [82]. The cost savings are calculated compared to the solvent market price, while the emission savings are calculated compared to the emissions emanating from the virgin solvent production process.

The results demonstrate the significant cost savings achieved by using pervaporation-based processes compared to traditional distillation methods and purchasing virgin solvents. These savings underscore the economic advantages of solvent recovery, making it a more sustainable and cost-effective option for industries. The results also demonstrate the environmental advantages of using pervaporation-based processes for solvent recovery. These processes generally result in lower CO_2_ emissions compared to traditional distillation methods and purchasing virgin solvents, highlighting their potential for reducing the industry’s carbon footprint.

All pervaporation-based processes show substantial cost savings compared to purchasing virgin solvents. The D-PV process for IPA saves €1568/t, and the PV process for ACN saves €1312/t. The recovery processes for THF (both PSD and D-PV-D) show the highest cost savings, with up to €1868/t. The savings for ACA are relatively lower compared to other solvents due to the lower market price of ACA. The VD process for NMP shows significant savings of €1381/t, while the D-PV process saves €1233/t. This indicates that a lot of improvements are required in the D-PV process to be able to recover NMP more cost-effectively than the VD process.

Traditional distillation methods (AD, ED, PSD, VD) generally show higher CO_2_ emissions compared to pervaporation-based processes. The AD process for IPA has emissions of 6.8 tonnes of CO_2_ per tonne of solvent, while D-PV has only 2.2 tonnes. Pervaporation-based processes (D-PV, PV, D-PV-D) show significant reductions in CO_2_ emissions. The PV process for ACN reduces emissions to 0.9 tonnes of CO_2_ per tonne of solvent, compared to 5.3 tonnes for ED. The D-PV-D process for THF shows a notable reduction in emissions (1.9 tonnes of CO_2_ per tonne of solvent) compared to the virgin solvent (5.9 tonnes). Some processes show minimal or no savings in CO_2_ emissions. The D-PV process for NMP has emissions of 3.8 tonnes of CO_2_ per tonne of solvent, which is slightly lower than the virgin solvent emissions (4.1 tonnes).

## 4. Conclusions

This study presented quantitative insights on the economic and environmental benefits of replacing the traditional distillation processes with pervaporation-based processes for five industrially relevant solvents, namely isopropanol, acetonitrile, tetrahydrofuran, acetic acid, and n-methyl-2-pyrrolidone. For the IPA-water system, the D-PV process leads to a 42% reduction in the LCOS, mainly from a significant reduction in operating costs (energy cost) compared to AD. Similarly, for the ACN-water system, the standalone PV process has moderate capital costs and the lowest operating costs, resulting in a 39% LCOS reduction, making it the most cost-effective for ACN dehydration. The hybrid D-PV-D process is the most cost-effective option for THF dehydration, with a 4% LCOS reduction compared to PSD. For the ACA-water system, the standalone PV process shows a 9% increase over the benchmark ED process due to the highest capital cost resulting from the pervaporation module. Among pervaporation-based processes, only the standalone PV process can outperform the benchmark ED process under specific conditions. For the NMP-water system, pervaporation-based processes (D-PV, D-PV-D, and PV) have higher LCOS due to high costs, with D-PV being the most interesting and standalone PV the least interesting. None of these processes shows lower LCOS than the benchmark VD process, but suitable conditions (permeate flux and membrane module price) could potentially lower the LCOS.

The CO_2_ emissions reduction is mainly due to decreased steam and cooling water use. For IPA-water, ACN-water, and THF-water systems, all pervaporation-based cases show significant CO_2_ reductions compared to the benchmarks, with standalone PV being the most effective. In the ACA-water system, standalone PV achieves an 82% CO_2_ reduction compared to the benchmark ED case. For the NMP-water system, standalone PV results in a 65% CO_2_ reduction, while D-PV and D-PV-D show increased emissions due to higher energy use.

The study highlights significant cost and environmental benefits of pervaporation-based processes over traditional distillation and virgin solvent purchase, making solvent recovery more sustainable and cost-effective. These processes generally lower CO_2_ emissions, reducing the industry’s carbon footprint. The results support replacing, debottlenecking, or intensifying traditional distillation-based solvent recovery in chemical, pharmaceutical, and related industries, benefiting both industries and society by reducing energy consumption and emissions.

## Figures and Tables

**Table 1:** Solvent-water systems and their benchmark processes.

Solvent/Water System	Benchmark Process	Pervaporation-Based Processes	Azeotrope Composition	Azeotrope Boiling Point	Source/Reference
Isopropanol (IPA)	Azeotropic distillation (AD)	D-PV, D-PV-D, and PV	12.3 wt.%	80.4 °C	[15]
Acetonitrile (ACN)	Extractive distillation (ED)	D-PV, D-PV-D, and PV	17.8 wt.%	76.5 °C	[43]
Tetrahydrofuran (THF)	Pressure-swing distillation (PSD)	D-PV, D-PV-D, and PV	6.7 wt.%	64 °C	[45]
Acetic acid (ACA)	Extractive distillation (ED)	D-PV, D-PV-D, and PV	Does not form an azeotrope		[44]
N-Methyl-2-pyrrolidone (NMP)	Vacuum distillation (VD)	D-PV, D-PV-D, and PV	Does not form an azeotrope		[46]

**Table 2:** Capital costs assumptions.

Item	Value
Base year	2024
Plant lifetime	25 yr
Chiller reference cost	€17,655 [63]
Chiller reference capacity	42 kW [63]
Vacuum pump reference cost	€60,600 ^a^
Vacuum pump reference capacity	18.5 kg/h∙kPa ^a^
Purchase cost (PC)	Aspen
Total installed cost (TIC)	PC × IF
Offsites (OS)	30% of TIC [62]
Design and Engineering (D&E)	30% of TIC + OS [62]
Contingency (X)	10% of TIC + OS [62]
Total plant cost (TPC)	TIC (1 + OS) (1 + D&E + X) [62]
WACC	4.8% ^b^

^a^ Vacuum pump modeled for the given size available in Aspen. ^b^ Discount rate = 9%, equity = 20%, debt = 80%, tax rate = 25%, interest rate = 5%. See Equation (S1).

**Table 3:** Economic assumptions on installation factors, scaling exponent, lifetime, and labor requirement.

Component	Installation Factor	Scaling Exponent	Lifetime	Labor
	-	-	(yr)	(Per Shift)
Column	4 ^a^	0.78 ^b^	25 ^f^	0.5 ^g^
Pump	4 ^a^	0.6 ^b^	25 ^f^	0.1 ^g^
Heater/cooler	3.5 ^a^	0.68 ^b^	25 ^f^	0.1 ^g^
Decanter	4 ^a^	0.57 [64]	25 ^f^	0.2 ^g^
Membrane	2 [15]	1 ^c^	5 ^e^	0.04 ^e^
Vacuum pump	1.7 [65]	0.75 ^b^	25 [66]	0.1 ^g^
Chiller	1.6 ^b^	0.6 ^d^	20 [67]	0.1 ^g^

^a^ [62]; ^b^ [68]; ^c^ modular; ^d^ assumption; ^e^ Pervatech; ^f^ average lifetime; ^g^ [69].

**Table 4:** Operation and maintenance cost assumptions.

Item	Value
Operating hours	8000 hr/yr
Insurance	1.5% of TPC [74]
Maintenance	2.5% of TPC [15]
Operating labor	2 persons [69]
Labor salary	60,000 €/yr/person [70]
Natural gas	€34/MWh [75]
Steam @ 8.75 bar	€35/t [71]
Steam @ 39.5 bar	€37/t [71]
Steam @ 85 bar	€36/t [71]
Electricity	€60/MWh [76]
Cooling water	€0.32/m^3^ [74]
Waste disposal cost	€3.2/m^3^ [72,73]
Membrane life	5 years ^a^
Benzene	€1135/t [77]
Ethylene glycol	€620/t [78]
Adiponitrile	€1650/t [79]

^a^ Membrane lifetime was obtained from Pervatech.

**Table 5:** CO_2_ emission intensity of utilities and chemicals.

Utility	CO_2_ Emission Intensity (t-CO_2_eq/Unit)
Electricity	0.107/MWh [80]
Steam	0.61/t [81]
Cooling water	0.03/t [81]
Benzene	1.76/t [81]
Ethylene glycol	3.09/t [82]
Adiponitrile	4.42/t [82]

**Table 6:** Experimental operating conditions and water/solvent separation factor.

Solvent	Experimental Feed Temperature (°C)	Vacuum Pressure (mbar)	Water Content Feed (wt.%)	Separation Factor (α)
IPA	114.7	18.7	3.5	977
NMP	127.8	20.9	4.2	485
THF	108.4	18.8	4.8	414
ACA	120.6	18.6	3.9	117
ACN	105.5	20.4	4.7	85

**Table 7:** Average fluxes and permeate quality.

Solvent	Cases	Inlet Water	Outlet Water	Avg. Water Flux	Solvent Flux	Permeate Quality
		wt.%	wt.%	kg/h/m^2^	kg/h/m^2^	wt.%
IPA	D-PV	15%	0.5%	8.00	0.1	98.8%
	D-PV-D	15%	5%	21.50	0.1	99.5%
	PV	50%	0.5%	25.84	0.1	99.6%
ACN	D-PV	25%	0.5%	14.71	1.46	91.0%
	D-PV-D	25%	5%	29.44	1.0	96.7%
	PV	50%	0.5%	28.15	1.12	96.2%
THF	D-PV	15%	0.5%	4.60	0.3	93.9%
	D-PV-D	15%	2%	17.21	0.2	98.9%
	PV	50%	0.5%	18.95	0.2	99.0%
ACA	D-PV	70%	0.5%	15.17	0.3	98.1%
	D-PV-D	70%	5%	22.59	0.3	98.7%
	PV	50%	0.5%	8.85	0.3	96.7%
NMP	D-PV	90%	0.5%	21.82	0.01	100.0%
	D-PV-D	90%	5%	25.35	0.01	100.0%
	PV	50%	0.5%	7.44	0.08	98.9%

**Table 8:** Technical performance metrics and utilities consumption.

Solvent	Cases	Recovery Efficiency	Membrane Area	Electricity	Steam	Energy Intensity	Cooling Water
		wt.%	m^2^	MWh/yr	t/yr	MWh/t	m^3^/yr
IPA	AD	99.7%	-	0 *	14,082	2.0	619,021
	D-PV	99.6%	11	164	5914	0.9	173,323
	D-PV-D	99.8%	4	163	11,557	1.7	466,523
	PV	99.6%	19	884	6059	1.1	421
ACN	ED	99.5%	-	0 *	11,371	1.6	477,193
	D-PV	96.7%	12	317	6095	1.0	168,369
	D-PV-D	98.7%	6	308	8715	1.3	308,245
	PV	96.0%	18	909	5735	1.1	419
THF	PSD	99.5%	-	4	7523	1.1	305,067
	D-PV	98.9%	20	170	3796	0.6	89,862
	D-PV-D	99.8%	5	166	4856	0.7	155,467
	PV	99.0%	27	892	5680	1.0	434
ACA	ED	100.0%	-	0 *	10,977	1.5	455,297
	D-PV	98.0%	33	885	13,418	2.1	387,159
	D-PV-D	98.7%	22	884	14,092	2.2	420,756
	PV	96.6%	58	889	5501	1.0	483
NMP	VD	99.5%	-	5 **	5565	0.8	233,934
	D-PV	100.0%	23	74	12,443	1.8	253,194
	D-PV-D	100.0%	20	881	12,525	2.0	255,327
	PV	98.9%	68	886	5698	1.0	496

* Electricity consumption in pumps was neglected due to operation at near atmospheric pressure ** Electricity consumption for the vacuum pump.

**Table 9:** Annualized capital and operating costs.

Solvent	Cases	CAPEX	OPEX	LCOS	% Reduction *
		€/yr	€/yr	€/t	%
IPA	AD	186,552	931,188	279	-
	D-PV	175,221	474,710	162	42%
	D-PV-D	263,075	835,940	274	2%
	PV	223,530	470,634	173	38%
ACN	ED	220,347	810,685	258	-
	D-PV	185,104	490,601	174	33%
	D-PV-D	168,546	635,619	203	21%
	PV	213,637	398,227	158	39%
THF	PSD	84,361	542,503	157	-
	D-PV	257,659	396,105	164	−5%
	D-PV-D	160,534	440,249	150	4%
	PV	292,166	416,969	178	−14%
ACA	ED	226,541	819,131	261	-
	D-PV	462,733	949,685	358	−38%
	D-PV-D	411,492	986,851	352	−36%
	PV	598,064	496,934	282	−9%
NMP	VD	84,846	391,570	119	-
	D-PV	297,525	775,616	267	−124%
	D-PV-D	307,563	841,745	286	−140%
	PV	692,039	530,526	307	−158%

* % Reduction compared to respective benchmark processes.

**Table 10:** Annual CO_2_ emissions and emission intensity.

Solvent	Cases	Emissions	Emission Intensity	% Reduction *
		tCO_2_/yr	tCO_2_/t	%
IPA	AD	27,161	6.8	-
	D-PV	8825	2.2	67%
	D-PV-D	21,063	5.3	22%
	PV	3803	0.9	86%
ACN	ED	21,254	5.3	-
	D-PV	8803	2.3	57%
	D-PV-D	14,597	3.7	31%
	PV	3608	0.9	82%
THF	PSD	13,741	3.4	-
	D-PV	5030	1.3	62%
	D-PV-D	7644	1.9	43%
	PV	3573	0.9	73%
ACA	ED	20,412	5.1	-
	D-PV	19,894	5.0	1%
	D-PV-D	21,313	5.4	−5%
	PV	3465	0.9	82%
NMP	VD	10,413	2.6	-
	D-PV	15,194	3.8	−45%
	D-PV-D	15,394	3.8	−47%
	PV	3585	0.9	65%

* % Reduction compared to respective benchmark processes.

## Data Availability

The original contributions presented in this study are included in the article/Supplementary Materials. Further inquiries can be directed to the corresponding authors.

## Supplementary Materials

The following supporting information can be downloaded at: https://www.mdpi.com/article/10.3390/membranes15120367/s1, Tables S1–S20: Stream conditions of all process configurations (AD, ED, PSD, VD, D-PV, D-PV-D, PV) across five solvent–water systems (IPA, ACN, THF, ACA, NMP); Tables S21–S40: Equipment cost data for each configuration and solvent system; Tables S41–S45: Column specifications in distillation and hybrid setups; Table S46: Interpretation of the COPCO index; Table S47: Price and CO2 intensity of virgin solvents. Figure S1: Visual interpretation of the COPCO index; Figures S2–S6: Capital cost breakdown per configuration and solvent; Figures S7–S11: Sensitivity analysis of LCOS to key input variables across solvent systems.
