# Supplementary material for: Advancing Solvent Dehydration with Innovative HybSi® AR Membranes: Economic and Environmental Benefits of Pervaporation
Source: Membranes (Basel). 2025 Dec 1;15(12):367. doi: 10.3390/membranes15120367 (PMC12735060; doi:10.3390/membranes15120367)
Supplement: Supplementary file 1 [file membranes-15-00367-s001.zip › membranes-3949909-supplementary.pdf]

Supplementary Material

For manuscript

Advancing Solvent Dehydration with Innovative HybSi® AR Membranes: Economic and Environmental Benefits of Pervaporation

Mohammed Nazeer Khan [1,*], Elmar Boorsma [2], Pieter Vandezande [1,*], Ilse Lammerink [2], Rob de Lange [2,3], Anita Buekenhoudt [1] and Miet Van Dael [1,4]

1 Unit Materials & Chemistry (MatCh), Flemish Institute for Technological Research (VITO), Boeretang 200, 2400 Mol, Belgium.
2 Pervatech B.V, Rijssen, The Netherlands.
3 Aswyn, Engelse Schans 12, 7137 SE Lievelde, The Netherlands.
4 Hasselt University, Centre for Environmental Sciences (CMK), Agoralaan, 3590 Diepenbeek, Belgium.

*Corresponding authors: pieter.vandezande@vito.be; mohammednazeer.khan@vito.be

S1. Annualized capital costs

The formula for the weighted average cost of capital (WACC) is described in equation [S1]. The WACC is the average cost of capital, taking into account the different sources of capital that a firm uses, and was estimated to be 4.8%. This value is based on equity ratio = 20%, debt ratio = 80%, discount rate = 9%, tax rate = 25% (Belgium), and interest rate = 5%.

$$WACC = (Equity * Discount\ rate) + \big(Debt * Interest\ rate * (1 - Tax\ rate)\big) \quad \text{(S1)}$$

The capital costs were annualized using the equation [S2].

$$Annualized\ cost = \frac{Total\ capital\ cost\ over\ plant\ lifetime}{\frac{1-(1+WACC)^{-Plant\ lifetime}}{WACC}} \quad \text{(S2)}$$

S2. Stream data

Table S1. Stream conditions of azeotropic distillation process for IPA-H_2O system.

Stream	Temperature	Mass flow rate	IPA	H_2O	C_6H_6
	°C	kg/hr	wt.%	wt.%	wt.%
1	20	1000	50.0%	50.0%	0.0%
2	80	716	86.5%	13.5%	0.0%
3	99	499	0.2%	99.8%	0.0%
4	62	1365	18.6%	11.4%	70.1%
5	82	501	99.5%	0.5%	0.0%
6	60	10	18.6%	7.6%	73.8%
7	81	215	56.2%	43.8%	0.0%
8	20	1150	11.5%	5.4%	83.2%
9	20	0.015	0.0%	0.0%	100.0%

Table S2. Stream conditions of D-PV process for IPA-H_2O system.

Stream	Temperature	Pressure	Mass flow rate	IPA	H_2O
	°C	bar	kg/hr	wt.%	wt.%
1	20	1	1000	50.0%	50.0%
2	80	1	587	85.0%	15.0%
3	99	1	413	0.2%	99.8%
4	130	6	587	85.0%	15.0%
5	130	6	498	99.5%	0.5%
6	17	0.02	87	1.2%	98.8%

Table S3. Stream conditions of D-PV-D process for IPA-H_2O system.

Stream	Temperature	Pressure	Mass flow rate	IPA	H_2O
	°C	bar	kg/hr	wt.%	wt.%
1	20	1	1000	50.0%	50.0%
2	80	1	587	85.0%	15.0%
3	99	1	413	0.2%	99.8%
4	130	6	960	86.5%	13.5%

5	130	6	874	95.0%	5.0%
6	17	0.02	86	0.5%	99.5%
7	80	1	372	88.9%	11.1%
8	82	1	501	99.5%	0.5%

Table S4. Stream conditions of PV process for IPA-H_2O system.

Stream	Temperature	Pressure	Mass flow rate	IPA	H_2O
	°C	bar	kg/hr	wt.%	wt.%
1	20	1	1000	50.0%	50.0%
2	130	6	1000	50.0%	50.0%
3	130	6	501	99.5%	0.5%
4	17	0.02	499	0.4%	99.6%

Table S5. Stream conditions of extractive distillation process for ACN-H_2O system.

Stream	Temperature	Mass flow rate	ACN	H_2O	$C_2H_6O_2$
	°C	kg/hr	wt.%	wt.%	wt.%
1	20	1000	50.0%	50.0%	0.0%
2	81	500	99.5%	0.5%	0.0%
3	114	1650	0.2%	30.2%	69.7%
4	99	500	0.5%	99.5%	0.0%
5	197	1150	0.0%	0.0%	100.0%
6	100	1150	0.0%	0.0%	100.0%
7	20	0.09	0.0%	0.0%	100.0%

Table S6. Stream conditions of D-PV process for ACN-H_2O system.

Stream	Temperature	Pressure	Mass flow rate	ACN	H_2O
	°C	bar	kg/hr	wt.%	wt.%
1	20	1	1000	50.0%	50.0%
2	76	1	666	75.0%	25.0%
3	99	1	334	0.2%	99.8%
4	130	6	666	75.0%	25.0%
5	130	6	486	99.5%	0.5%
6	-1	0.02	180	8.8%	91.2%

Table S7. Stream conditions of D-PV-D process for ACN-H_2O system.

Stream	Temperature	Pressure	Mass flow rate	ACN	H_2O
	°C	bar	kg/hr	wt.%	wt.%
1	20	1	1000	50.0%	50.0%
2	76	1	666	75.0%	25.0%
3	99	1	334	0.2%	99.8%
4	130	6	1124	81.2%	18.8%

5	130	6	954	95.0%	5.0%
6	8	0.02	170	3.3%	96.7%
7	77	1	458	90.1%	9.9%
8	81	1	496	99.5%	0.5%

Table S8. Stream conditions of PV process for ACN-H_2O system.

Stream	Temperature	Pressure	Mass flow rate	ACN	H_2O
	°C	bar	kg/hr	wt.%	wt.%
1	20	1	1000	50.0%	50.0%
2	130	6	1000	50.0%	50.0%
3	130	6	483	99.5%	0.5%
4	6	0.02	517	3.8%	96.2%

Table S9. Stream conditions of pressure-swing distillation process for THF-H_2O system.

Stream	Temperature	Pressure	Mass flow rate	THF	H_2O
	°C	bar	kg/hr	wt.%	wt.%
1	20	1	1000	50.0%	50.0%
2	72	2.5	2135	71.2%	28.8%
3	65	1.05	1635	92.8%	7.2%
4	105	1.35	500	0.5%	99.5%
5	65	8	1635	92.8%	7.2%
6	130	7	1135	89.8%	10.2%
7	140	7.3	500.00	99.5%	0.5%

Table S10. Stream conditions of D-PV process for THF-H_2O system.

Stream	Temperature	Pressure	Mass flow rate	THF	H_2O
	°C	bar	kg/hr	wt.%	wt.%
1	20	1	1000	50.0%	50.0%
2	64	1	588	85.0%	15.0%
3	100	1	412	0.0%	100.0%
4	130	7.5	588	85.0%	15.0%
5	130	7.5	497	99.5%	0.5%
6	-1	0.02	91	6.1%	93.9%

Table S11. Stream conditions of D-PV-D process for THF-H_2O system.

Stream	Temperature	Pressure	Mass flow rate	THF	H_2O
	°C	bar	kg/hr	wt.%	wt.%
1	20	1	1000	50.0%	50.0%
2	64	1	588	85.0%	15.0%
3	100	1	412	0.0%	100.0%
4	130	7.5	1051	90.0%	10.0%

5	130	7.5	964	98.0%	2.0%
6	11	0.02	87	1.4%	98.6%
7	63	1	463	96.4%	3.6%
8	65	1	501	99.5%	0.5%

Table S12. Stream conditions of PV process for THF-H_2O system.

Stream	Temperature	Pressure	Mass flow rate	THF	H_2O
	°C	bar	kg/hr	wt.%	wt.%
1	20	1	1000	50.0%	50.0%
2	130	7.5	1000	50.0%	50.0%
3	130	7.5	497	99.5%	0.5%
4	12	0.02	503	1.0%	99.0%

Table S13. Stream conditions of extractive distillation process for ACA-H_2O system.

Stream	Temperature	Mass flow rate	ACA	H_2O	$C_6H_8N_2$
	°C	kg/hr	wt.%	wt.%	wt.%
1	20	1000	50.0%	50.0%	0.0%
2	100	500	0.2%	99.8%	0.0%
3	135	1102	45.3%	0.1%	54.6%
4	117	500	99.5%	0.2%	0.3%
5	287	602	0.2%	0.0%	99.7%
6	100	602	0.2%	0.0%	99.7%
7	20	1.63	0.0%	0.0%	100.0%

Table S14. Stream conditions of D-PV process for ACA-H_2O system.

Stream	Temperature	Pressure	Mass flow rate	ACA	H_2O
	°C	bar	kg/hr	wt.%	wt.%
1	20	1	1000	50.0%	50.0%
2	101	1	712	30.0%	70.0%
3	117	1	288	99.5%	0.5%
4	130	3	712	30.0%	70.0%
5	130	3	205	99.5%	0.5%
6	18	0.02	507	1.9%	98.1%

Table S15. Stream conditions of D-PV-D process for ACA-H_2O system.

Stream	Temperature	Pressure	Mass flow rate	ACA	H_2O
	°C	bar	kg/hr	wt.%	wt.%
1	20	1	1000	50.0%	50.0%
2	101	1	712	30.0%	70.0%
3	117	1	288	99.5%	0.5%
4	130	3	918	43.6%	56.4%

5	130	3	414	95.0%	5.0%
6	18	0.02	504	1.3%	98.7%
7	111	1	206	90.4%	9.6%
8	117	1	208	99.5%	0.5%

Table S16. Stream conditions of PV process for ACA-H_2O system.

Stream	Temperature	Pressure	Mass flow rate	ACA	H_2O
	°C	bar	kg/hr	wt.%	wt.%
1	20	1	1000	50.0%	50.0%
2	130	3	1000	50.0%	50.0%
3	130	3	486	99.5%	0.5%
4	18	0.02	514	3.3%	96.7%

Table S17. Stream conditions of vacuum distillation process for NMP-H_2O system.

Stream	Temperature	Pressure	Mass flow rate	NMP	H_2O
	°C	bar	kg/hr	wt.%	wt.%
1	20	1	1000	50.0%	50.0%
2	80	1	1000	50.0%	50.0%
3	46	0.1	500	0.5%	99.5%
4	112	0.1	500	99.5%	0.5%

Table S18. Stream conditions of D-PV process for NMP-H_2O system.

Stream	Temperature	Pressure	Mass flow rate	NMP	H_2O
	°C	bar	kg/hr	wt.%	wt.%
1	20	1	1000	50.0%	50.0%
2	100	1	553	10.0%	90.0%
3	187	1	447	99.5%	0.5%
4	130	3	553	10.0%	90.0%
5	130	3	55	99.5%	0.5%
6	30	0.02	498	0.0%	100.0%

Table S19. Stream conditions of D-PV-D process for NMP-H_2O system.

Stream	Temperature	Pressure	Mass flow rate	NMP	H_2O
	°C	bar	kg/hr	wt.%	wt.%
1	20	1	1000	50.0%	50.0%
2	100	1	553	10.0%	90.0%
3	187	1	447	99.5%	0.5%
4	130	3	560	10.6%	89.4%
5	130	3	63	95.0%	5.0%
6	17	0.02	498	0.0%	100.0%
7	75	1	7	60.2%	39.8%

8	187	1	55	99.5%	0.5%

Table S20. Stream conditions of PV process for NMP-H_2O system.

Stream	Temperature	Pressure	Mass flow rate	NMP	H_2O
	°C	bar	kg/hr	wt.%	wt.%
1	20	1	1000	50.0%	50.0%
2	130	2.5	1000	50.0%	50.0%
3	130	2.5	497	99.5%	0.5%
4	18	0.02	503	1.1%	98.9%

S3. Economic data

Table S21. Equipment cost of IPA azeotropic distillation.

Equipment	Capacity (kg/hr)	Cost (€)
Column 1	1215	127,500
Column 2	1866	139,700
Condenser	1364	14,600
Decanter	1375	21,900
Column 3	225	66,600

Table S22. Equipment cost of IPA D-PV process.

Equipment	Capacity (kg/hr)	Cost (€)
Column 1	1,000	92,900
Pump	587	6,200
Heater	587	9,900
Pervaporation	587	227,466
Interstage heater	87	9,900
Condenser	87	10,900
Vacuum pump	1.3 kg/hr·kPa	8,055
Chiller	64 kW	22,702

Table S23. Equipment cost of IPA D-PV-D process.

Equipment	Capacity (kg/hr)	Cost (€)
Column 1	1,000	100,700
Pump	960	6,000
Heater	960	11,300
Pervaporation	960	83,986
Interstage heater	86	11,200
Condenser	86	11,600
Vacuum pump	1.1 kg/hr·kPa	7,767
Chiller	63.6 kW	22,649

Column 2	873.6	293,300

Table S24. Equipment cost of IPA PV process.

Equipment	Capacity (kg/hr)	Cost (€)
Pump	1,000	6,000
Heater	1,000	9,900
Pervaporation	1,000	405,884
Interstage heater	499	12,500
Condenser	499	16,600
Vacuum pump	1.3 kg/hr·kPa	8,331
Chiller	369.6 kW	65,101

Table S25. Equipment cost of ACN extractive distillation.

Equipment	Capacity (kg/hr)	Cost (€)
Column 1	2150	265,100
Column 2	1650	158,800
Cooler	1150	11,300

Table S26. Equipment cost of ACN D-PV process.

Equipment	Capacity (kg/hr)	Cost (€)
Column 1	1,000	80,300
Pump	666	5,900
Heater	666	9,700
Pervaporation	666	256,631
Interstage heater	164	10,700
Condenser	180	10,900
Vacuum pump	1.3 kg/hr·kPa	8,105
Chiller	129 kW	34,621

Table S27. Equipment cost of ACN D-PV-D process.

Equipment	Capacity (kg/hr)	Cost (€)
Column 1	1,000	80,300
Pump	1124	6,000
Heater	1124	9,900
Pervaporation	1124	116,942
Interstage heater	164	10,700
Condenser	170	170
Vacuum pump	1.2 kg/hr·kPa	7,843
Chiller	125 kW	33,936
Column 2	954	94,200

Table S28. Equipment cost of ACN PV process.

Equipment	Capacity (kg/hr)	Cost (€)
Pump	1,000	5,594

Heater	1,000	10,700
Pervaporation	1,000	385,990
Interstage heater	497.59	12,500
Condenser	517.41	14,000
Vacuum pump	1.3 kg/hr·kPa	8,303
Chiller	380 kW	66,210

Table S29. Equipment cost of THF pressure-swing distillation.

Equipment	Capacity (kg/hr)	Cost (€)
Pump 1	1000	5,230
Column 1	2135	75,900
Column 2	1635	79,800
Pump 2	1635	6,200

Table S30. Equipment cost of THF D-PV process.

Equipment	Capacity (kg/hr)	Cost (€)
Column 1	1,000	83,900
Pump	588	5,800
Heater	588	9,900
Pervaporation	588	417,052
Interstage heater	86	9,900
Condenser	91	10,700
Vacuum pump	1.3 kg/hr·kPa	8,347
Chiller	66 kW	23,244

Table S31. Equipment cost of THF D-PV-D process.

Equipment	Capacity (kg/hr)	Cost (€)
Column 1	1,000	83,900
Pump	1051	5,900
Heater	1051	9,900
Pervaporation	1051	104,603
Interstage heater	86	9,900
Condenser	87	9,900
Vacuum pump	1.2 kg/hr·kPa	7,815
Chiller	64 kW	22,828
Column 2	964	92,700

Table S32. Equipment cost of THF PV process.

Equipment	Capacity (kg/hr)	Cost (€)

Pump	1,000	5,727
Heater	1,000	9,900
Pervaporation	1,000	557,148
Interstage heater	497.51	12,500
Condenser	502.76	16,600
Vacuum pump	1.4 kg/hr·kPa	8,531
Chiller	373 kW	65,448

Table S33. Equipment cost of ACA extractive distillation.

Equipment	Capacity (kg/hr)	Cost (€)
Column 1	1602	343,100
Column 2	1102	93,300
Cooler	602	10,500

Table S34. Equipment cost of ACA D-PV process.

Equipment	Capacity (kg/hr)	Cost (€)
Column 1	1,000	203,900
Pump	712	5,600
Heater	712	9,700
Pervaporation	712	702,369
Interstage heater	498	12,500
Condenser	507	16,500
Vacuum pump	1.4 kg/hr·kPa	8,705
Chiller	370 kW	65,184

Table S35. Equipment cost of ACA D-PV-D process.

Equipment	Capacity (kg/hr)	Cost (€)
Column 1	1,000	203,900
Pump	918	5,700
Heater	918	9,700
Pervaporation	918	462,503
Interstage heater	498	12,500
Condenser	504	16,500
Vacuum pump	1.3 kg/hr·kPa	8,409
Chiller	370 kW	65,132
Column 2	414	116,500

Table S36. Equipment cost of ACA PV process.

Equipment	Capacity (kg/hr)	Cost (€)
Pump	1,000	6,000
Heater	1,000	9,900
Pervaporation	1,000	1,220,705
Interstage heater	497.57	12,500
Condenser	514.44	16,600

Vacuum pump	1.5 kg/hr·kPa	9,237
Chiller	371 kW	65,296

Table S37. Equipment cost of NMP vacuum distillation.

Equipment	Capacity (kg/hr)	Cost (€)
Column 1	1000	157,300
Heater	1000	9,700
Vacuum system	0.5 kg/hr·kPa	4,238

Table S38. Equipment cost of NMP D-PV process.

Equipment	Capacity (kg/hr)	Cost (€)
Column 1	1,000	109,900
Pump	553	5,600
Heater	553	9,700
Pervaporation	553	479,009
Interstage heater	497	12,500
Condenser	498	9,700
Vacuum pump	1.3 kg/hr·kPa	8,430
Chiller	26 kW	13,244

Table S39. Equipment cost of NMP D-PV-D process.

Equipment	Capacity (kg/hr)	Cost (€)
Column 1	1,000	109,900
Pump	560	5,600
Heater	560	9,700
Pervaporation	560	412,121
Interstage heater	497	12,500
Condenser	498	19,600
Vacuum pump	1.3 kg/hr·kPa	8,340
Chiller	369 kW	65,030
Column 2	63	48,400

Table S40. Equipment cost of NMP PV process.

Equipment	Capacity (kg/hr)	Cost (€)
Pump	1,000	0
Heater	1,000	10,700

Pervaporation	1,000	1,419,363
Interstage heater	497.51	12,500
Condenser	502.86	19,600
Vacuum pump	1.6 kg/hr·kPa	9,417
Chiller	370 kW	65,156

S4. Column specifications

Table S41. Column specifications of IPA cases.

Process		Column 1	Column 2	Column 3
AD	Stages	10	20	10
	Feed stage	8	8	4
	Reflux ratio	2.48	0.96	0.52
D-PV	Stages	10	-	-
	Feed stage	8	-	-
	Reflux ratio	0.67	-	-
D-PV-D	Stages	10	28	-
	Feed stage	8	13	-
	Reflux ratio	0.67	3.8	-

Table S42. Column specifications of ACN cases.

Process		Column 1	Column 2
ED	Stages	26	10
	Feed/entrainer stage	18/4	5
	Reflux ratio	0.90	0.27
D-PV	Stages	5	-
	Feed stage	3	-
	Reflux ratio	0.16	-
D-PV-D	Stages	5	10
	Feed stage	3	6
	Reflux ratio	0.2	0.76

Table S43. Column specifications of THF cases.

Process		Column 1	Column 2
PSD	Stages	6	6
	Feed stage	3	3
	Reflux ratio	0.03	0.02
D-PV	Stages	7	-
	Feed stage	5	-

	Reflux ratio	0.10	-
D-PV-D	Stages	7	10
	Feed stage	5	5
	Reflux ratio	0.10	0.53

Table S44. Column specifications of ACA cases.

Process		Column 1	Column 2
ED	Stages	30	6
	Feed/entrainer stage	12/4	3
	Reflux ratio	0.69	0.01
D-PV	Stages	20	-
	Feed stage	10	-
	Reflux ratio	0.67	-
D-PV-D	Stages	20	14
	Feed stage	10	5
	Reflux ratio	0.7	0.5

Table S45. Column specifications of NMP cases.

Process		Column 1	Column 2
VD	Stages	7	-
	Feed stage	4	-
	Reflux ratio	0.02	-
D-PV	Stages	5	-
	Feed stage	4	-
	Reflux ratio	0.13	-
D-PV-D	Stages	5	3
	Feed stage	4	2
	Reflux ratio	0.13	0.1

S5. COPCO index interpretation

Table S46. Interpretation of COPCO index

Scenario	Cost difference (€/t-IPA)	CO_2 difference ($t\text{-}CO_2$/t-IPA)	Cost per tonne of CO_2 (€/$t\text{-}CO_2$)	Interpretation
1	positive	positive	+X+	€ saved per CO_2 saved

2	negative	positive	-X+	€ increased per CO_2 saved
3	positive	negative	+X-	€ saved per CO_2 emitted
4	negative	negative	-X-	€ increased per CO_2 emitted

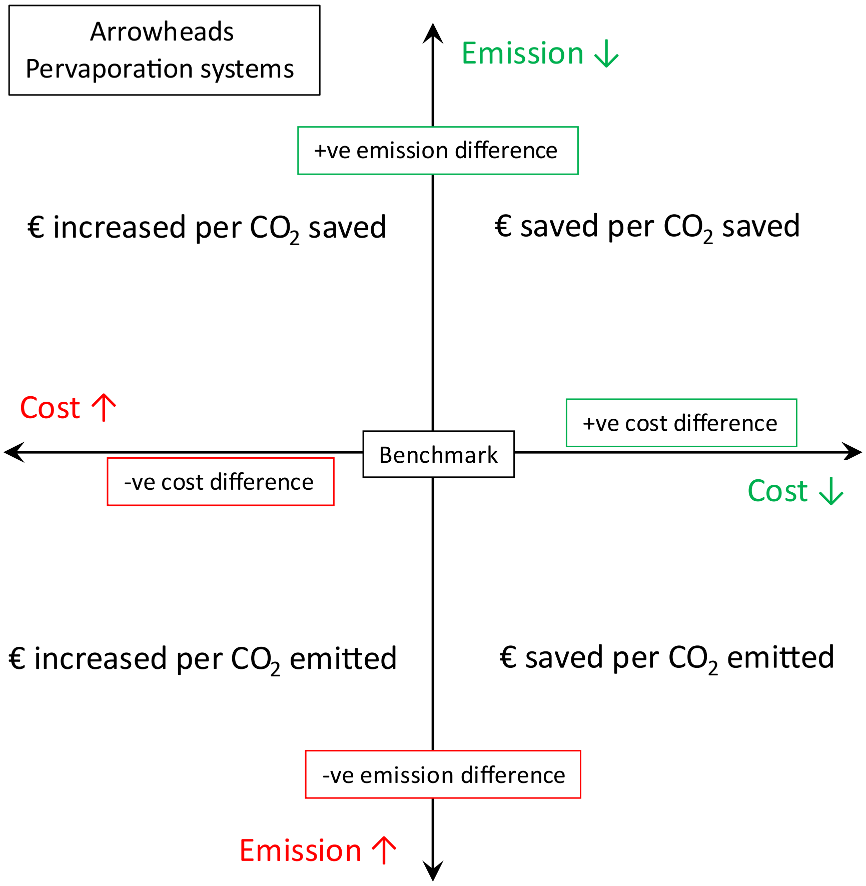

Figure S1. Visual interpretation of COPCO index.

S6. Economic results

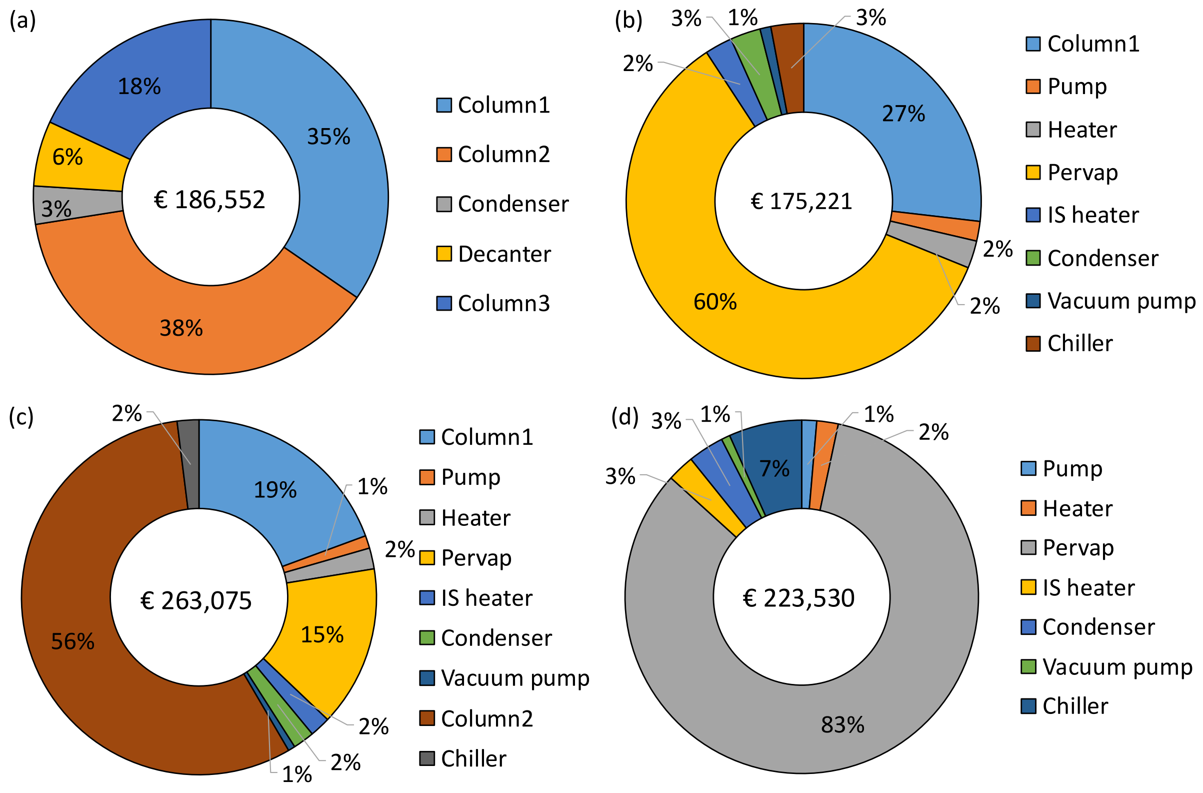

Figure S2. Annualized capital cost breakdown of IPA system (a) AD, (b) D-PV, (c) D-PV-D, and (d) PV.

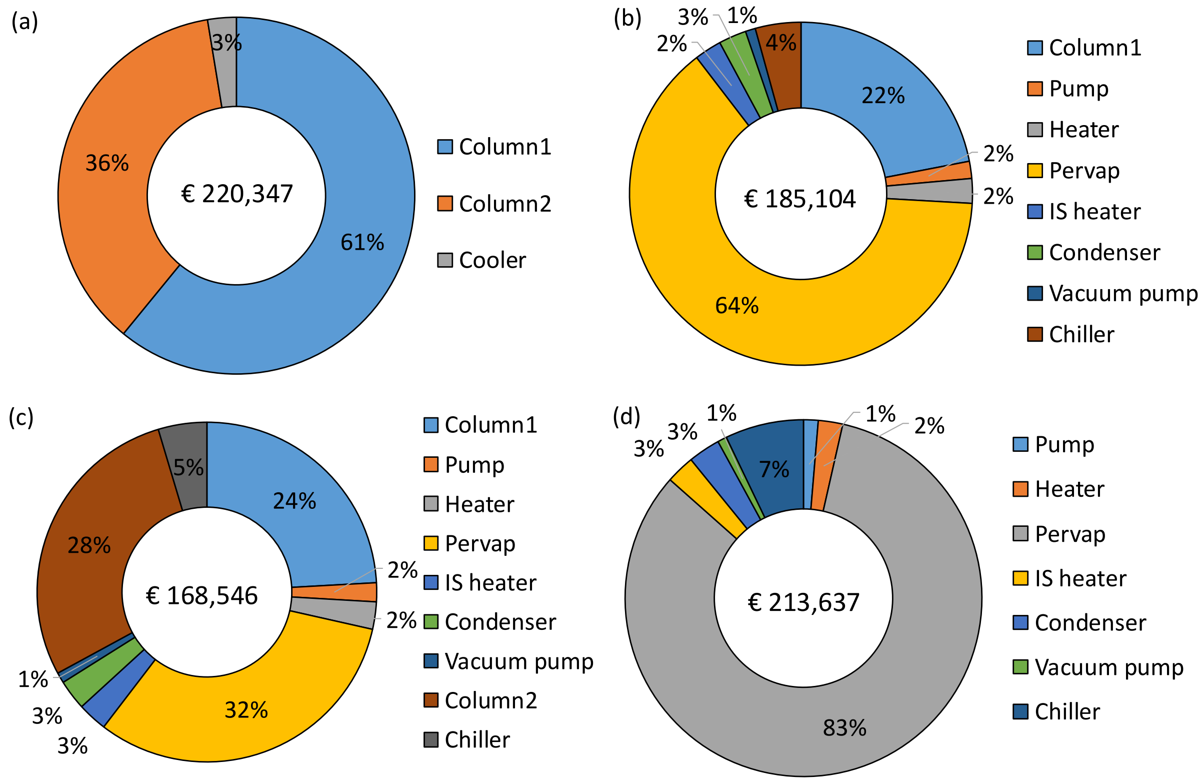

Figure S3. Annualized capital cost breakdown of ACN system (a) AD, (b) D-PV, (c) D-PV-D, and (d) PV.

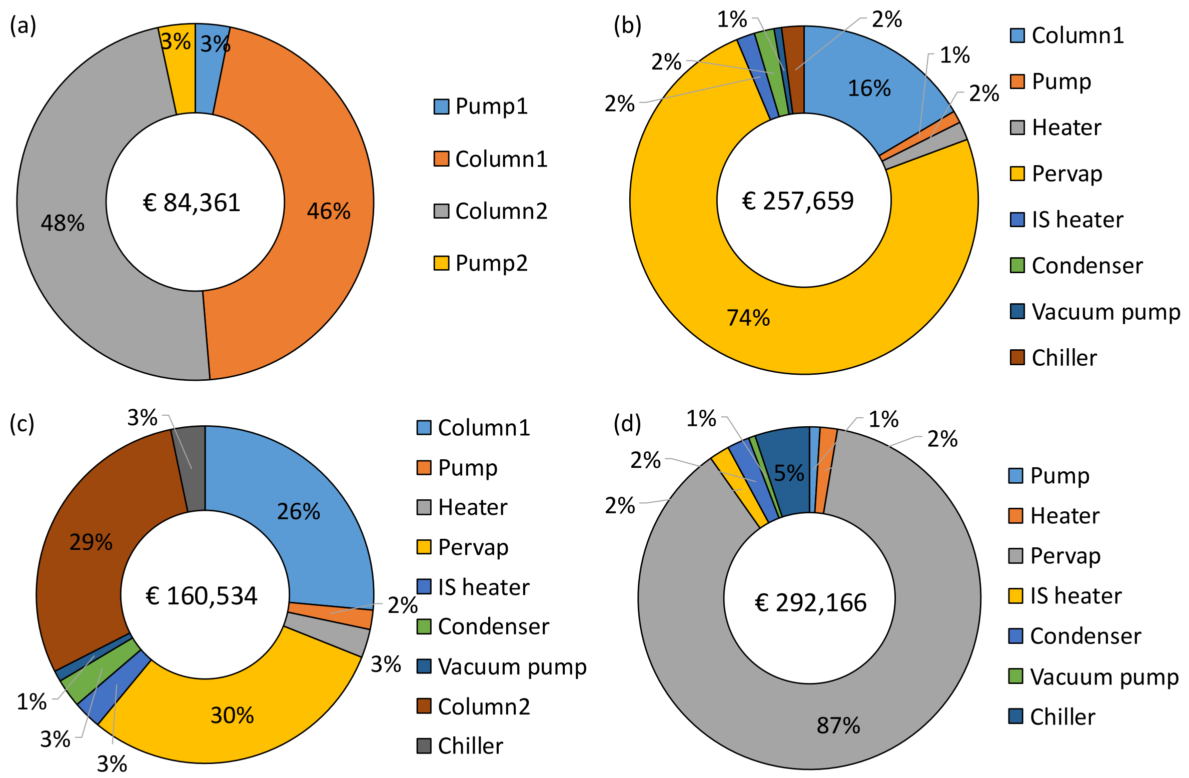

Figure S4. Annualized capital cost breakdown of THF system (a) AD, (b) D-PV, (c) D-PV-D, and (d) PV.

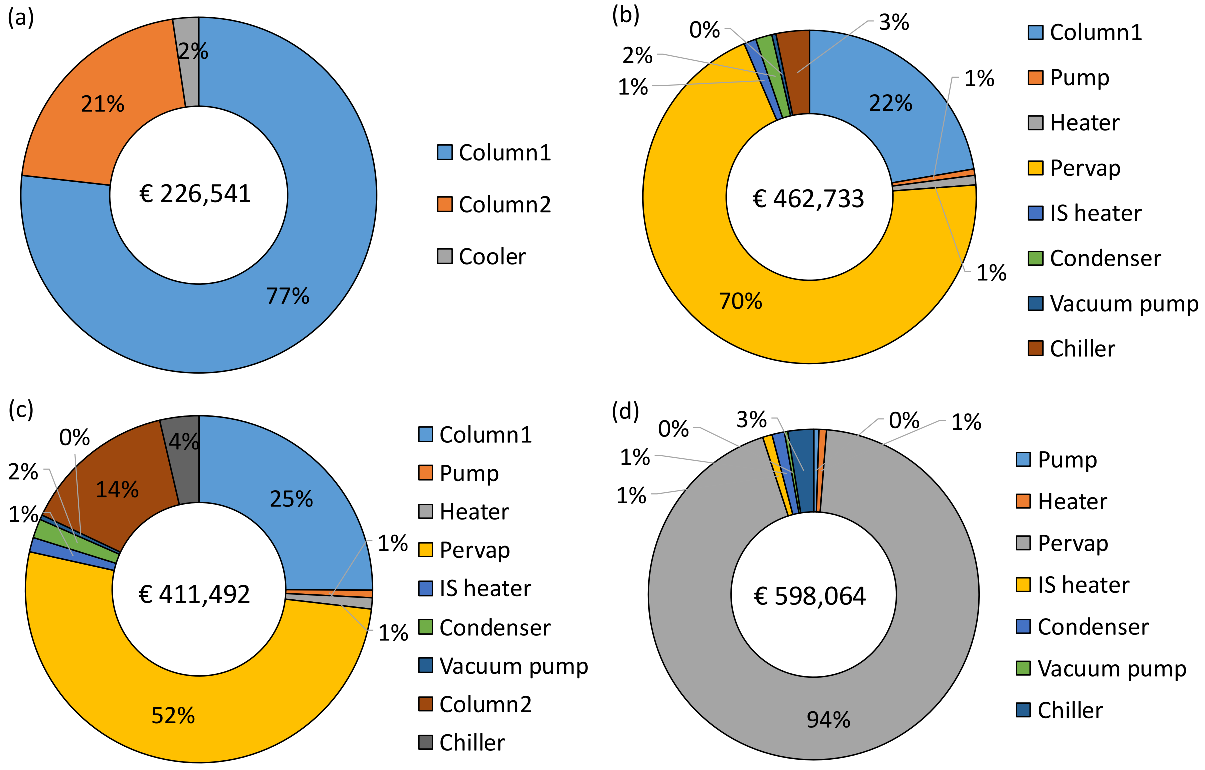

Figure S5. Annualized capital cost breakdown of ACA system (a) AD, (b) D-PV, (c) D-PV-D, and (d) PV.

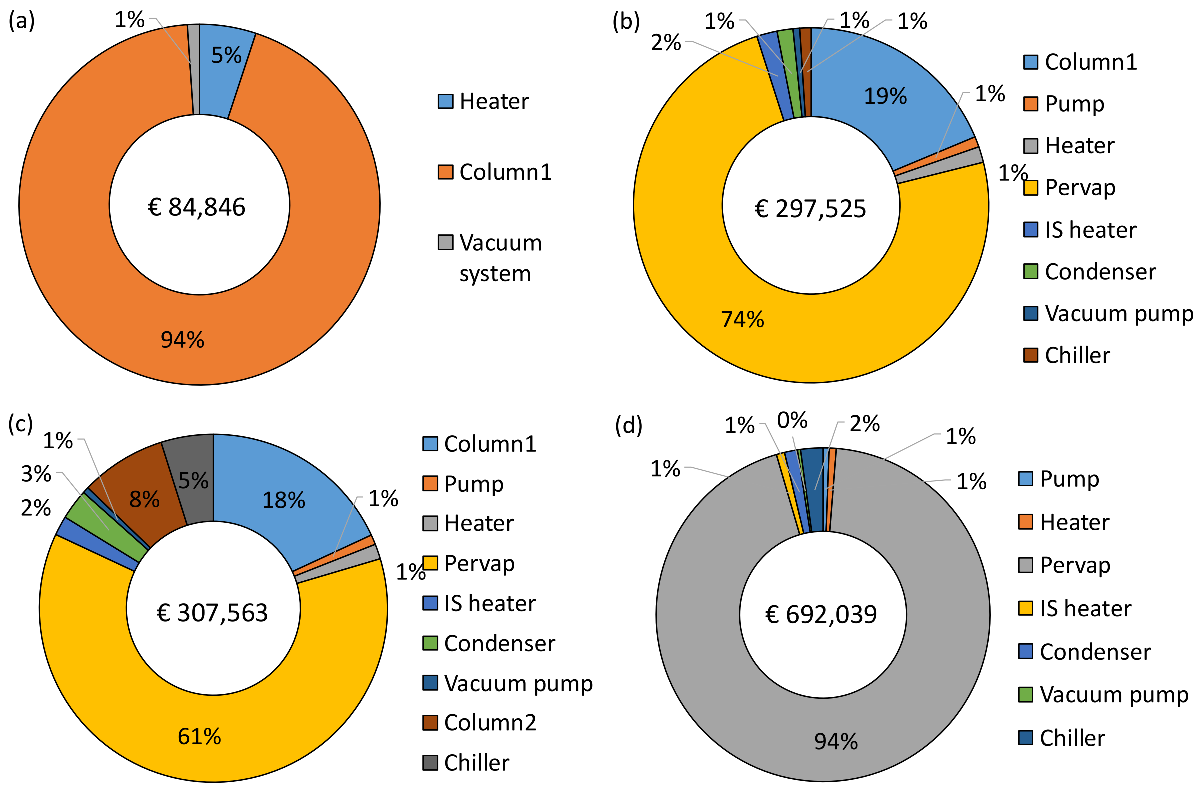

Figure S6. Annualized capital cost breakdown of NMP system (a) AD, (b) D-PV, (c) D-PV-D, and (d) PV.

S7. Sensitivity analysis

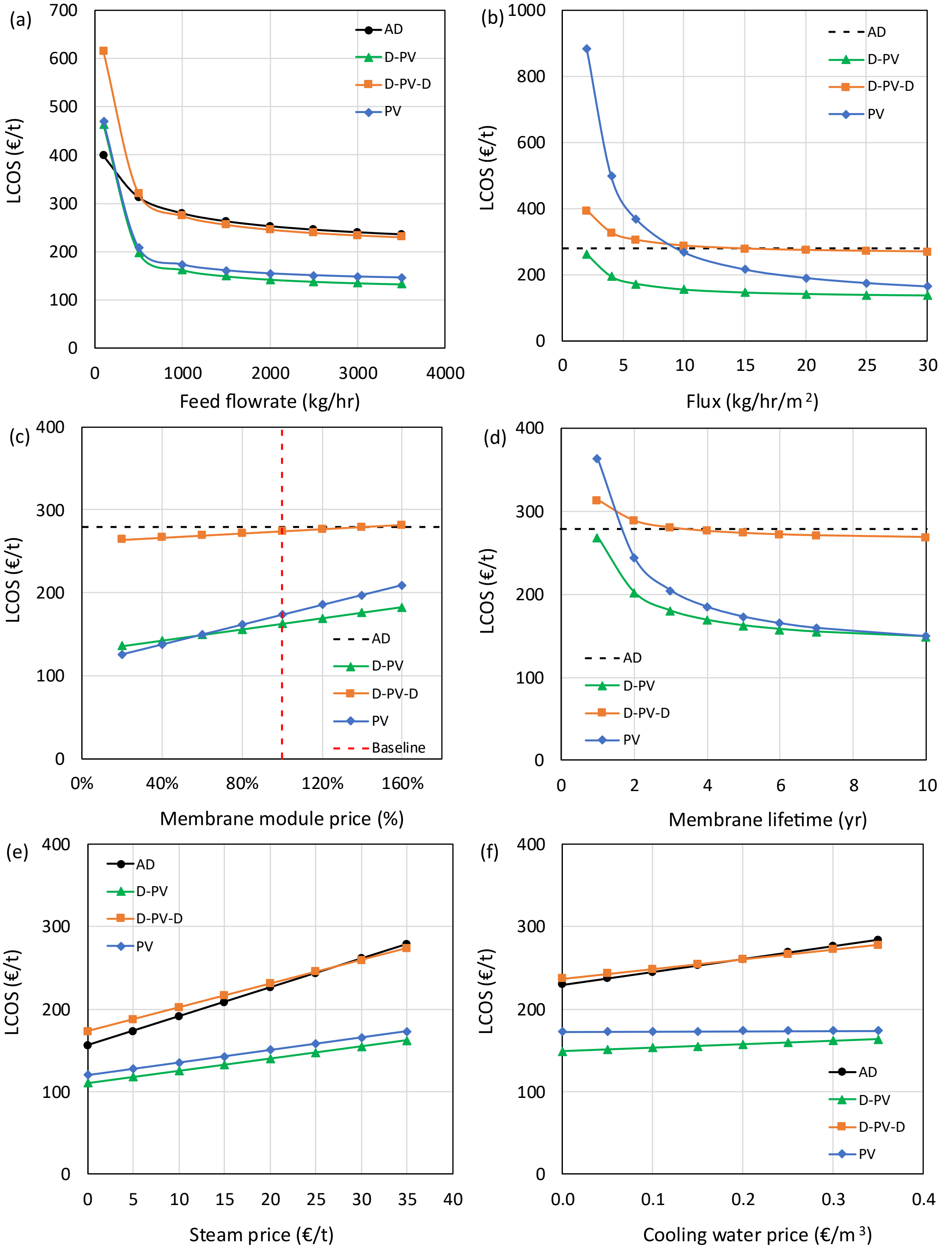

Figure S7. Sensitivity of LCOS in IPA-H_2O system with respect to (a) feed flow rate, (b) permeate flux, (c) membrane module price, (d) membrane life, (e) steam price, and (f) cooling water price.

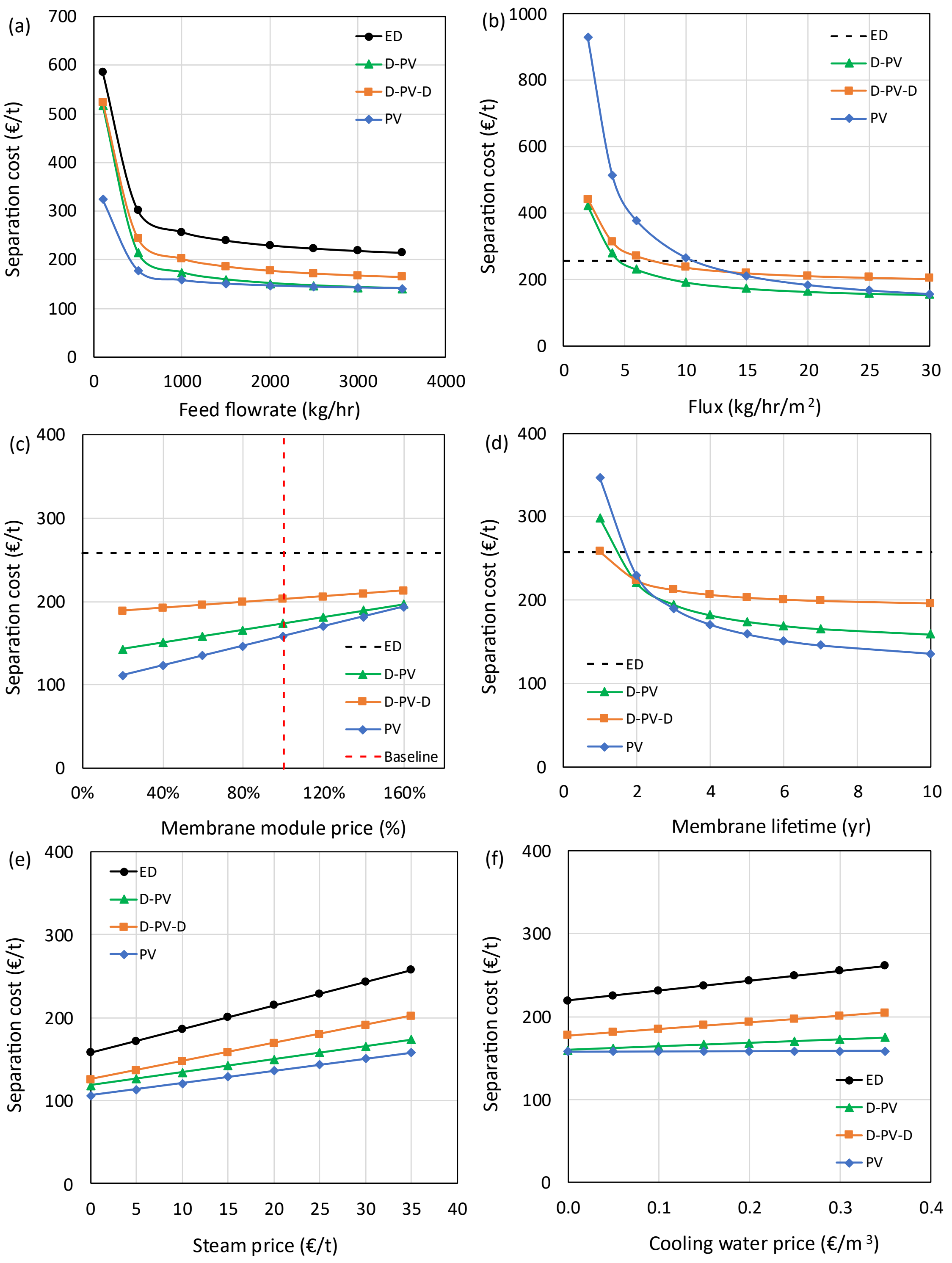

Figure S8. Sensitivity of LCOS in ACN-H_2O system with respect to (a) feed flow rate, (b) permeate flux, (c) membrane module price, (d) membrane life, (e) steam price, and (f) cooling water price.

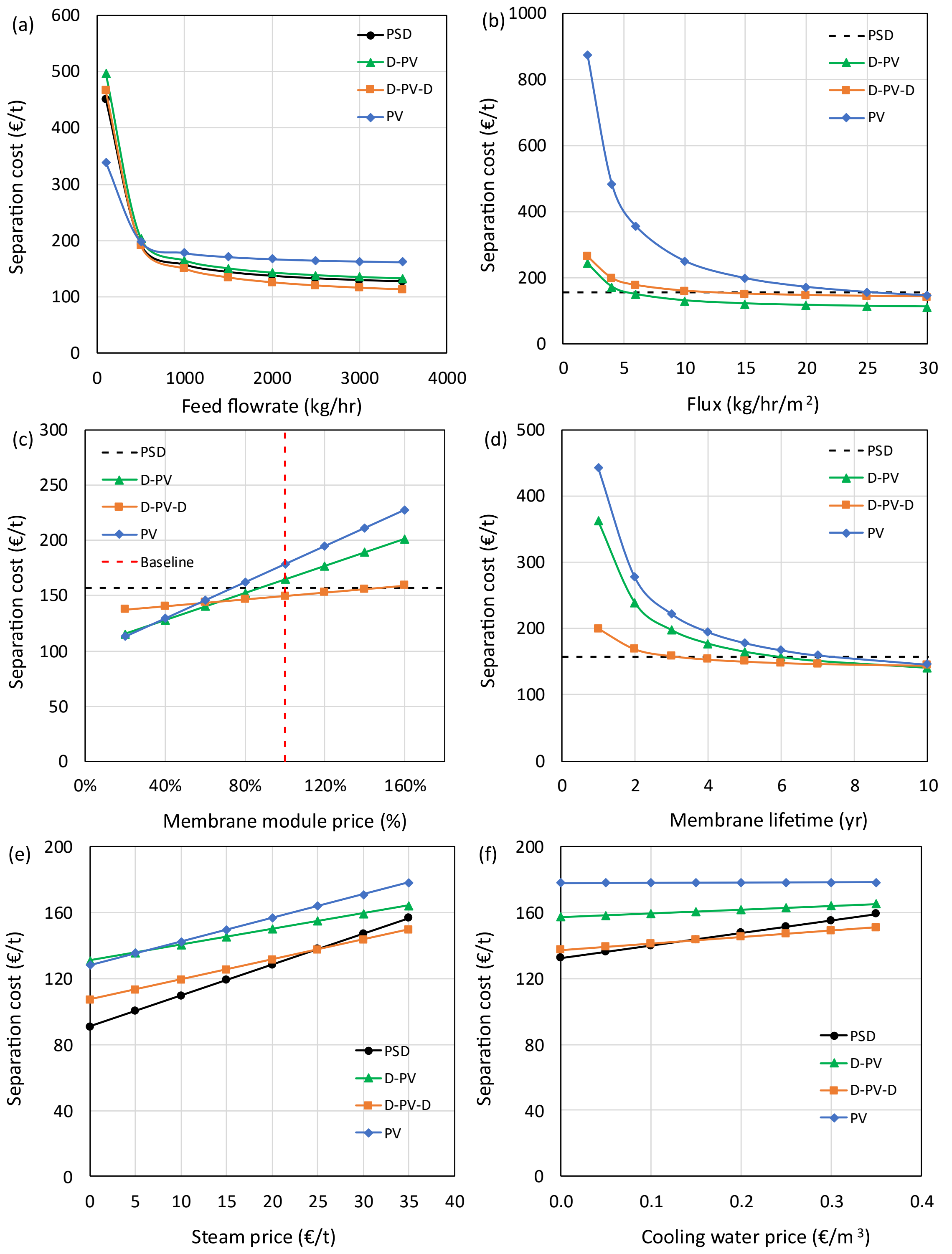

Figure S9. Sensitivity of LCOS in THF-H_2O system with respect to (a) feed flow rate, (b) permeate flux, (c) membrane module price, (d) membrane life, (e) steam price, and (f) cooling water price.

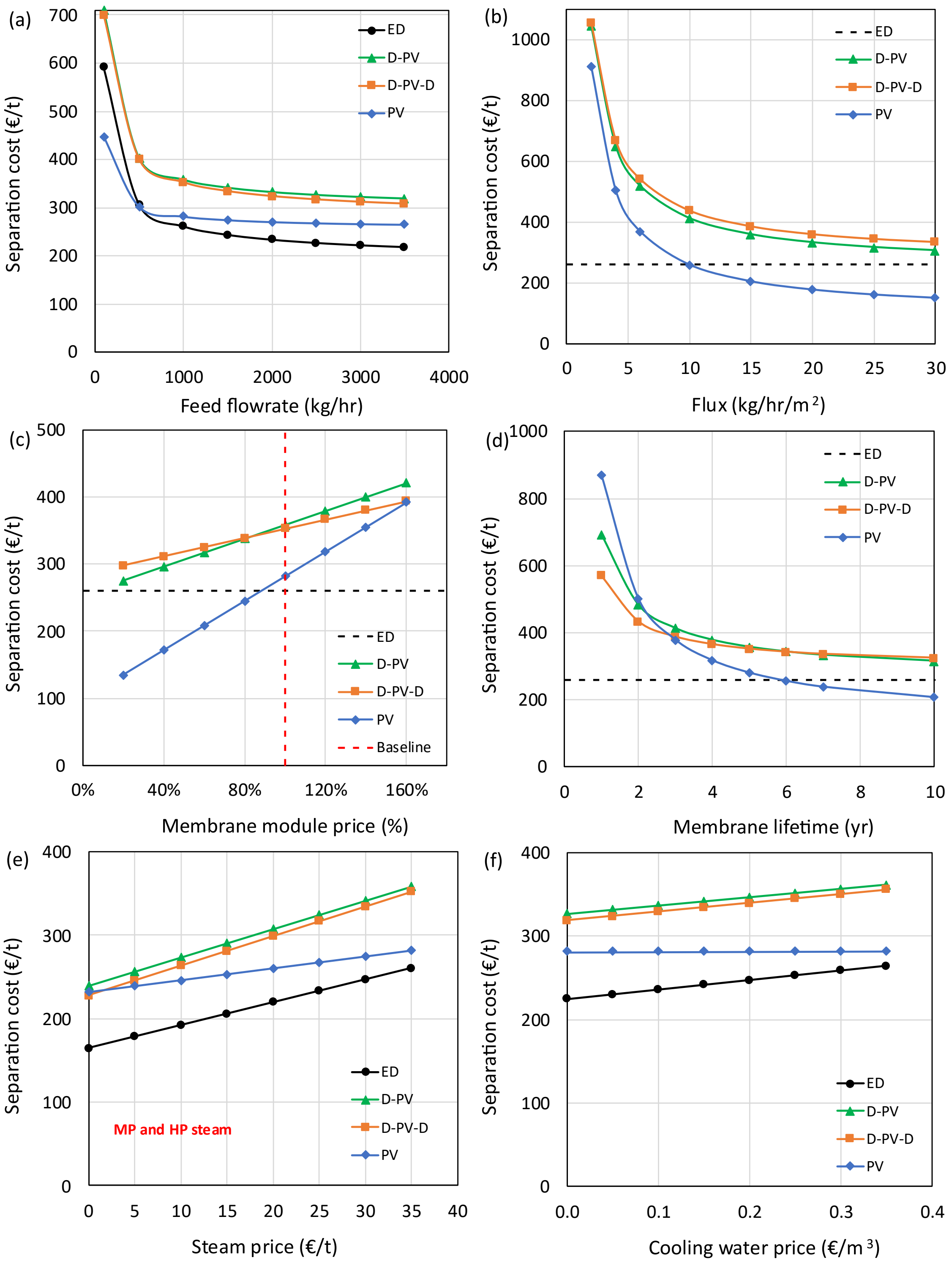

Figure S10. Sensitivity of LCOS in ACA-H_2O system with respect to (a) feed flow rate, (b) permeate flux, (c) membrane module price, (d) membrane life, (e) steam price, and (f) cooling water price.

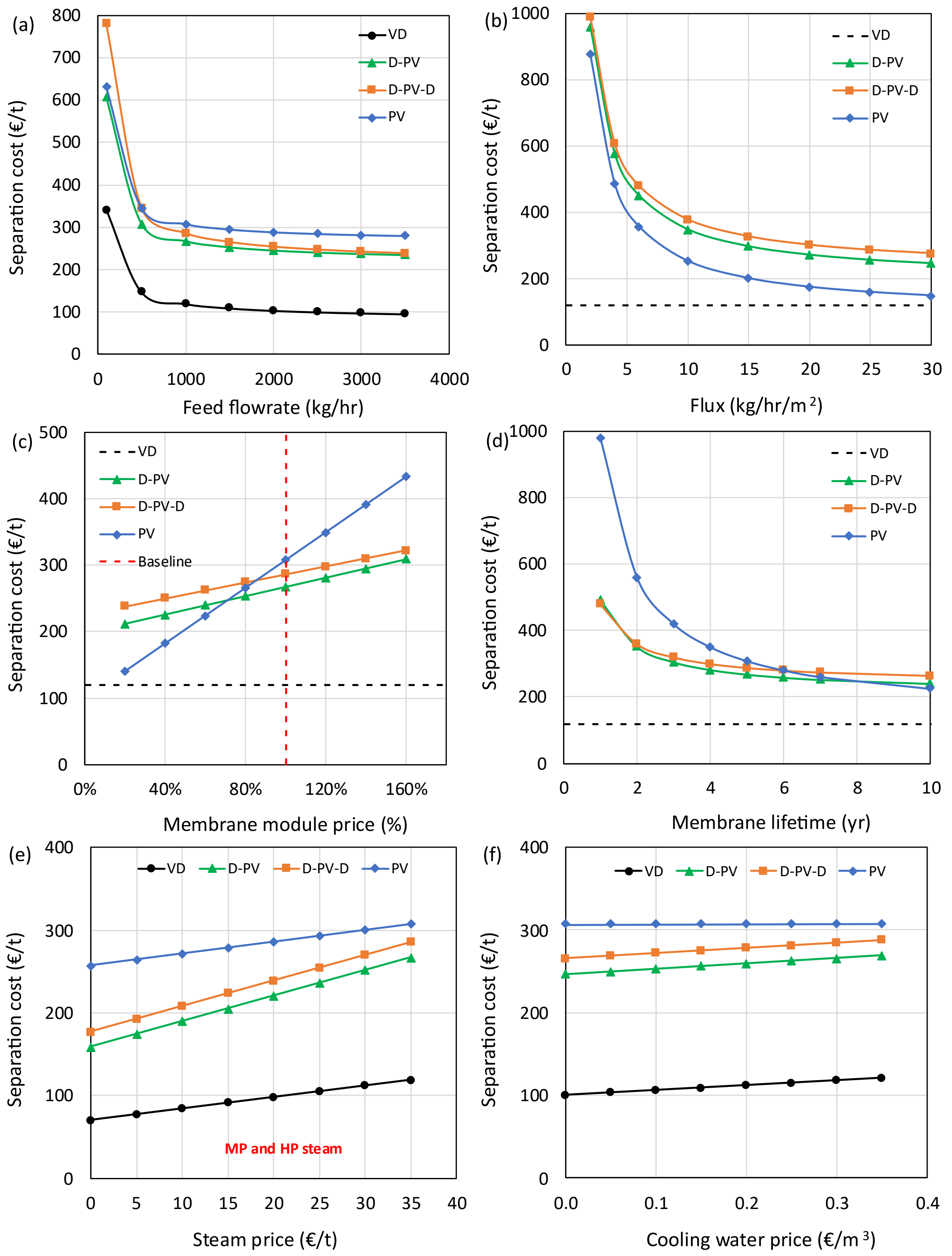

Figure S11. Sensitivity of LCOS in NMP-H_2O system with respect to (a) feed flow rate, (b) permeate flux, (c) membrane module price, (d) membrane life, (e) steam price, and (f) cooling water price.

S8. Cost and emissions of virgin solvents

Table S47. Market price and emission intensity of virgin solvents.

Solvent	Market price (€/t)	Emission intensity (t-CO_2/t)

Isopropanol	€1730/t [1]	1.95 [2]
Acetonitrile	€1470/t [3]	3.18 [2]
Tetrahydrofuran	€2018/t [4]	5.88 [2]
Acetic acid	€715/t [5]	1.65 [2]
N-Methyl-2-pyrrolidone	€1500/t [6]	4.05 [2]

S9. References

1. Chemanalyst N Propanol Prices Available online: https://www.chemanalyst.com/Pricing-data/n-propanol-1182 (accessed on 24 September 2024).
2. Wernet, G.; Bauer, C.; Steubing, B.; Reinhard, J.; Moreno-Ruiz, E.; Weidema, B. The Ecoinvent Database Version 3 (Part I): Overview and Methodology. *Int. J. Life Cycle Assess.* **2016**, *21*, 1218–1230, doi:10.1007/s11367-016-1087-8.
3. Chemanalyst Acetonitrile Available online: https://www.chemanalyst.com/Pricing-data/acetonitrile-1105.
4. Chemanalyst Tetrahydrofuran (THF) Available online: https://www.chemanalyst.com/Pricing-data/tetrahydrofuran-1213 (accessed on 14 October 2024).
5. Chemanalyst Acetic Acid Available online: https://www.chemanalyst.com/Pricing-data/acetic-acid-9.
6. Made-in-China N-Methyl-2-Pyrrolidone Price Available online: https://www.made-in-china.com/products-search/hot-china-products/NMP_Price.html.
